# Risk of Potentially Preventable Hospitalizations After SARS-CoV-2 Infection

**DOI:** 10.1001/jamanetworkopen.2024.5786

**Published:** 2024-04-10

**Authors:** Diana J. Govier, Meike Niederhausen, Yumie Takata, Alex Hickok, Mazhgan Rowneki, Holly McCready, Valerie A. Smith, Thomas F. Osborne, Edward J. Boyko, George N. Ioannou, Matthew L. Maciejewski, Elizabeth M. Viglianti, Amy S. B. Bohnert, Ann M. O’Hare, Theodore J. Iwashyna, Denise M. Hynes

**Affiliations:** 1 Center to Improve Veteran Involvement in Care, Veterans Affairs (VA) Portland Health Care System, Portland, Oregon; 2 Oregon Health & Science University–Portland State University School of Public Health, Portland; 3 College of Health, Oregon State University, Corvallis; 4 Center of Innovation to Accelerate Discovery and Practice Transformation, VA Durham Health Care System, Durham, North Carolina; 5 Department of Medicine, Duke University, Durham, North Carolina; 6 Department of Population Health Sciences, Duke University School of Medicine, Durham, North Carolina; 7 VA Palo Alto Health Care System, Palo Alto, California; 8 Department of Radiology, Stanford University School of Medicine, Stanford, California; 9 Seattle Epidemiologic Research and Information Center, VA Puget Sound Health Care System, Seattle, Washington; 10 Center of Innovation for Veteran-Centered and Value-Driven Care, VA Puget Sound Health Care System, Seattle, Washington; 11 Division of Gastroenterology, Department of Medicine, University of Washington, Seattle; 12 VA Center for Clinical Management Research, VA Ann Arbor Health Care System, Ann Arbor, Michigan; 13 Division of Pulmonary and Critical Care Medicine, Department of Internal Medicine, University of Michigan, Ann Arbor; 14 Department of Psychiatry, University of Michigan Medical School, Ann Arbor; 15 Hospital and Specialty Medicine Service, VA Puget Sound Health Care System, Seattle, Washington; 16 Division of Nephrology, Department of Medicine, University of Washington, Seattle; 17 School of Medicine, Johns Hopkins University, Baltimore, Maryland; 18 Department of Medicine, University of Michigan Medical School, Ann Arbor; 19 School of Public Health, Johns Hopkins University, Baltimore, Maryland; 20 Center for Quantitative Life Sciences, Oregon State University, Corvallis, Oregon; 21 School of Nursing, Oregon Health & Science University, Portland

## Abstract

**Importance:**

Research demonstrates that SARS-CoV-2 infection is associated with increased risk of all-cause hospitalization. However, no prior studies have assessed the association between SARS-CoV-2 and potentially preventable hospitalizations—that is, hospitalizations for conditions that can usually be effectively managed in ambulatory care settings.

**Objective:**

To examine whether SARS-CoV-2 is associated with potentially preventable hospitalization in a nationwide cohort of US veterans.

**Design, Setting, and Participants:**

This cohort study used an emulated target randomized trial design with monthly sequential trials to compare risk of a potentially preventable hospitalization among veterans with SARS-CoV-2 and matched comparators without SARS-CoV-2. A total of 189 136 US veterans enrolled in the Veterans Health Administration (VHA) who were diagnosed with SARS-CoV-2 between March 1, 2020, and April 30, 2021, and 943 084 matched comparators were included in the analysis. Data were analyzed from May 10, 2023, to January 26, 2024.

**Exposure:**

SARS-CoV-2 infection.

**Main Outcomes and Measures:**

The primary outcome was a first potentially preventable hospitalization in VHA facilities, VHA-purchased community care, or Medicare fee-for-service care. Extended Cox models were used to examine adjusted hazard ratios (AHRs) of potentially preventable hospitalization among veterans with SARS-CoV-2 and comparators during follow-up periods of 0 to 30, 0 to 90, 0 to 180, and 0 to 365 days. The start of follow-up was defined as the date of each veteran’s first positive SARS-CoV-2 diagnosis, with the same index date applied to their matched comparators.

**Results:**

The 1 132 220 participants were predominantly men (89.06%), with a mean (SD) age of 60.3 (16.4) years. Most veterans were of Black (23.44%) or White (69.37%) race. Veterans with SARS-CoV-2 and comparators were well-balanced (standardized mean differences, all <0.100) on observable baseline clinical and sociodemographic characteristics. Overall, 3.10% of veterans (3.81% of those with SARS-CoV-2 and 2.96% of comparators) had a potentially preventable hospitalization during 1-year follow-up. Risk of a potentially preventable hospitalization was greater among veterans with SARS-CoV-2 than comparators in 4 follow-up periods: 0- to 30-day AHR of 3.26 (95% CI, 3.06-3.46); 0- to 90-day AHR of 2.12 (95% CI, 2.03-2.21); 0- to 180-day AHR of 1.69 (95% CI, 1.63-1.75); and 0- to 365-day AHR of 1.44 (95% CI, 1.40-1.48).

**Conclusions and Relevance:**

In this cohort study, an increased risk of preventable hospitalization in veterans with SARS-CoV-2, which persisted for at least 1 year after initial infection, highlights the need for research on ways in which SARS-CoV-2 shapes postinfection care needs and engagement with the health system. Solutions are needed to mitigate preventable hospitalization after SARS-CoV-2.

## Key Points

**Question:**

Is infection with SARS-CoV-2 associated with an increased risk of potentially preventable hospitalization, and if so, how long does this association persist after infection?

**Findings:**

In this cohort study of 1 132 220 US veterans enrolled in the Veterans Health Administration, veterans with SARS-CoV-2 had 3 times greater risk of potentially preventable hospitalization than matched comparators without SARS-CoV-2 within 30 days after infection and more than 40% greater risk at 1 year.

**Meaning:**

These findings suggest that the persistently higher risk of potentially preventable hospitalization among veterans with SARS-CoV-2 infection may reflect difficulty meeting postinfection ambulatory health care needs in the broader context of the pandemic.

## Teaser

This cohort study examines the association between SARS-CoV-2 and the risk of potentially preventable hospitalization in Veterans Health Administration (VHA) facilities, VHA-purchased community care, and Medicare fee-for-service care among a national cohort of veterans enrolled in the VHA.

## Introduction

Delayed or inadequate treatment of ambulatory care–sensitive conditions (acute or chronic conditions that can be treated effectively through quality ambulatory care [ACSCs]) can result in hospitalization.^1^ Thus, hospitalizations for ACSCs (hereinafter referred to as potentially preventable hospitalizations) are widely recognized as an indicator of ambulatory care access and quality.^1,2,3,4^ They are also increasingly used as a measure of health system performance during public health emergencies.^5^

During the COVID-19 pandemic, health system capacity and pandemic-related restrictions caused disruptions to outpatient care delivery in the US, which may have hindered ACSC treatment and increased potentially preventable hospitalizations.^6,7,8,9,10,11,12^ Messaging early in the pandemic encouraging people to stay home when sick, as well as postinfection sequelae, may have compounded these risks among individuals with SARS-CoV-2.^13^ However, studies show potentially preventable hospitalizations declined in the US during the first year of the pandemic.^6,7^ However, with only contemporaneous pre–COVID-19 era comparison periods or groups,^6,7^ these studies were not designed to detect the effect of SARS-CoV-2 on potentially preventable hospitalizations. Regarding hospitalizations more generally, studies mostly report that SARS-CoV-2 is associated with an increased risk of all-cause hospitalization.^14,15,16,17^

Understanding the influence of SARS-CoV-2 infection on potentially preventable hospitalizations is necessary for evaluating whether patients’ postinfection health care needs are being met. We examined the association, during varying follow-up periods, between SARS-CoV-2 and the risk of potentially preventable hospitalization in Veterans Health Administration (VHA) facilities, VHA-purchased community care, and Medicare fee-for-service care among a national cohort of veterans enrolled in the VHA, the largest integrated national health system in the US. We additionally characterized the association between SARS-CoV-2 and potentially preventable hospitalizations during 3 pandemic waves and by selected clinical and sociodemographic factors.

## Methods

This cohort study was approved by the institutional review boards of the Ann Arbor, Michigan; Durham, North Carolina; Palo Alto, California; Portland, Oregon; and Puget Sound, Washington, VHA medical centers, which waived the informed consent requirement as this was a retrospective study of extant data in accordance with 45 CFR §46. This study followed the Strengthening the Reporting of Observational Studies in Epidemiology (STROBE) reporting guideline.

### Target Trial Approach and Study Participants

We emulated nested monthly sequential trials to assess the risk of potentially preventable hospitalization among VHA patients with SARS-CoV-2. Data from the Veterans Affairs (VA) COVID-19 Shared Data Resource (CSDR)^18^ were used to identify SARS-CoV-2 diagnoses between March 1, 2020, and April 30, 2021, a period during which health care facility–based testing was common and self-testing was rarer. The CSDR integrates a variety of data sources to provide patient-level information on VHA facility–, non-VHA facility–, and community-detected infections.^18^

Race and ethnicity were self reported by participants and were categorized as American Indian or Alaska Native, Asian, Black, Hispanic, Native Hawaiian or Other Pacific Islander, White, and multiple races or ethnicities. These data were collected in electronic health records and for descriptive purposes, because health care utilization including potentially preventable hospitalizations are known to vary by race and ethnicity.

Emulating the balance achieved through randomization (eTable 1 in Supplement 1), we a priori identified potential confounders associated with SARS-CoV-2 and clinical, functional, and economic outcomes.^19^ We then used VA electronic health record data to identify and create matched monthly cohorts composed of similar veterans with and without (hereinafter termed *comparators*) SARS-CoV-2 during the same month as their matched veteran’s documented infection (index date). Matched cohorts were created using a combination of exact matching and propensity score matching with replacement. Veterans without a history of SARS-CoV-2 in a given month could be matched to more than 1 veteran with SARS-CoV-2 and were eligible for matching in subsequent months until infected. Our matching procedure included 5 exact-matched variables and 37 propensity score–matched variables, including baseline sociodemographic, clinical, and health care use variables (eTable 2 in Supplement 1).^20^

Veterans were included if they had used VHA primary care during the 2 years prior to the index date or were enrolled in a VHA Patient-Aligned Care Team service. We excluded veterans who had missing age and missing or implausible weight or height data; resided outside the 50 US states or Washington, DC; died during baseline; or lacked a suitable match. Veterans with SARS-CoV-2 diagnoses (*International Statistical Classification of Diseases and Related Health Problems, Tenth Revision*, codes B97.29, U07.1, U09.9, J12.82, and Z86.16) in Medicare fee-for-service claims at least 15 days prior to their CSDR-documented infection were also excluded to avoid misclassification. For this analysis, we included up to 5 comparators nearest in propensity score to their matched veteran with SARS-CoV-2; 0.5% of the SARS-CoV-2 cohort had at least 1 but fewer than 5 matched comparators (eFigure 1 in Supplement 1 for a study flow diagram).

### Measures

Our primary exposure was SARS-CoV-2 infection, described above. Our primary outcome was the first potentially preventable hospitalization in either the VHA, VHA-purchased community care, or Medicare fee-for-service care.^21,22,23^ We identified potentially preventable hospitalizations using Agency for Healthcare Research & Quality prevention quality indicators (PQIs), which are defined based on the principal diagnosis of a hospital stay and have been validated for identifying hospitalizations for ACSCs.^1^ Outcomes included PQI overall and acute and chronic composites (eTable 3 in Supplement 1).^24^ The overall composite consists of the chronic composite (eg, diabetes with short or long-term complications, chronic obstructive pulmonary disease) and acute composite (eg, bacterial pneumonia, urinary tract infection). In primary analyses, hospitalizations that occurred on the same date as the index date or death were counted as outcomes.

Baseline sociodemographic, clinical, and health care use variables (eTable 2 in Supplement 1) were used to match veterans with SARS-CoV-2 to comparators and control for potential confounding in subgroup analyses. Additional variables used to identify strata for subgroup analyses included COVID-19 pandemic wave of index date (March 1 to June 30, 2020, July 1 to November 31, 2020, and December 1, 2020, to April 30, 2021),^25^ baseline Elixhauser rehospitalization risk score,^26^ residence in a primary care professional shortage area,^27^ and hospitalization at index date. Primary care professional shortage area data were obtained from the Health Resources & Services Administration.^27^ Variables for Medicare Advantage status at index date and long-term institutionalization (ie, inpatient stays >180 days)^3^ during the 2 years prior to or 1 year after the index date were used to identify veterans for inclusion or exclusion in sensitivity analyses.

### Statistical Analysis

Data were analyzed from May 10, 2023, to January 26, 2024. To assess exchangeability on observable data and descriptively examine outcomes between SARS-CoV-2 and comparator groups, we calculated frequencies and proportions, means and SDs, and standardized mean differences (SMDs).^28,29^ Cumulative incidence plots were used to assess overall trends in time to first potentially preventable hospitalization, with crossover infection among comparators and death treated as censoring events.

For our primary analysis, which we refer to herein as per-protocol 1 (PP1), we used extended Cox regression models^30^ and a stratified baseline hazard specification based on matched group to estimate the mean hazard ratio (HR) of overall, acute, and chronic composite potentially preventable hospitalizations during 4 cumulative follow-up periods: 0 to 30, 0 to 90, 0 to 180, and 0 to 365 days. We treated non–PQI-based (ie, nonpreventable) hospitalizations as a time-varying covariate,^31^ defined as a binary variable equal to 1 during intervals when a veteran was hospitalized and otherwise 0. Veterans who had not yet experienced an outcome or another censoring event were right censored at the end of each follow-up period. Crossover infection among comparators and death among infected and comparators were treated as censoring events. We used Hubert-White robust SEs to account for clustering on matched group.

We conducted a series of sensitivity analyses for the overall composite outcome. First, potentially preventable hospitalizations that occurred on the index date were eliminated, as these may have resulted in incidental and unrelated SARS-CoV-2 diagnoses. Second, because we did not have access to Medicare Advantage data, we excluded individual comparators with Medicare Advantage and matched groups in which the veteran with SARS-CoV-2 had Medicare Advantage (346 988 [30.65%]). Third, we excluded individual comparators who were institutionalized at baseline or follow-up and matched groups in which the veteran with SARS-CoV-2 was institutionalized at baseline or follow-up, as these individuals may have systematically different risk of SARS-CoV-2 and hospitalization (210 768 [18.62%]). Fourth, to understand risk during discrete vs cumulative intervals, we estimated models with discrete follow-up periods: 0 to 30, 31 to 90, 91 to 180, and 181 to 365 days. For this analysis, individuals no longer at risk for an outcome due to previous events, crossover infections, or death were excluded. Additionally, matched groups without at least 1 at-risk veteran with SARS-CoV-2 and 1 at-risk comparator were excluded. Last, we tested a second form of censoring, which we refer to herein as per-protocol 2 (PP2), in which we censored the entire matched group at the time of a crossover infection among comparators; deaths were censored individually as in PP1.

Using our PP1 approach, we conducted exploratory subgroup analysis based on age group (<65, 65-85, and ≥85 years) and sex (male or female). In addition, using our PP1 approach but without a stratified baseline hazard specification, we conducted subgroup analyses based on COVID-19 pandemic wave (March 1 to June 30, 2020, July 1 to November 30, 2020, and December 1, 2020, to April 30, 2021), baseline Elixhauser rehospitalization risk score tertile, baseline residence in a primary care professional shortage area (yes or no), and hospitalization at index date (yes or no). In subgroup models, we adjusted for variables with SMDs greater than 0.10, as some variables used for matching were less well balanced across strata. For our hospitalization-at-index subgroup analysis, outcome events that occurred on the same day as index date were excluded.

Statistical significance was determined a priori at α = .05 (2-sided). All analyses were conducted in R, version 4.3.1 (R Project for Statistical Computing).

## Results

### Cohort Characteristics

Our sample included 1 132 220 veterans, 189 136 (16.70%) of whom had SARS-CoV-2 between March 1, 2020, and April 30, 2021 (Table 1). Baseline characteristics were well-balanced between SARS-CoV-2 and comparator groups (all SMDs <0.10). Overall, the mean (SD) age at index date was 60.3 (16.4) years; 89.06% were male and 10.94% were female; the cohort had a mean (SD) Elixhauser rehospitalization risk score of 20.83 (22.73). In terms of self-reported race and ethnicity, 0.94% were American Indian or Alaska Native, 1.02% were Asian, 23.44% were Black, 9.70% were Hispanic, 0.94% were Native Hawaiian or Other Pacific Islander, 69.37% were White, and 1.07% were of multiple races or ethnicities. Most cohort members resided in urban areas (70.03%), and 24.11% in primary care professional shortage areas. Cohort members were well-connected with the VHA, with a mean (SD) of 8.31 (10.27) VHA primary care contacts (ie, face-to-face visits, telephone interactions, clinician time managing patient care) during the 2 years prior to index date.

**Table 1.** Sample Characteristics for SARS-CoV-2 and Matched Uninfected Comparators^a^

Variable^b^	Participant group			SMD
	All (N = 1 132 220)	SARS-CoV-2 infection (n = 189 136)	Comparator (n = 943 084)	
**Variables used in matching**					
Age, y				
<65	614 955 (54.31)	108 233 (57.22)	506 722 (53.73)	0.071
65-84	462 684 (40.87)	72 192 (38.17)	390 492 (41.41)	
≥85	54 581 (4.82)	8711 (4.61)	45 870 (4.86)	
Sex				
Male	1 008 374 (89.06)	168 395 (89.03)	839 979 (89.07)	0.001
Female	123 846 (10.94)	20 741 (10.97)	103 105 (10.93)	
Race^c^				
American Indian or Alaska Native	10 626 (0.94)	1785 (0.94)	8841 (0.94)	0.033
Asian	11 550 (1.02)	1928 (1.02)	9622 (1.02)	
Black	265 357 (23.44)	44 794 (23.68)	220 563 (23.39)	
Native Hawaiian or Other Pacific Islander	10 627 (0.94)	1771 (0.94)	8856 (0.94)	
White	785 391 (69.37)	129 834 (68.65)	655 557 (69.51)	
Multiple	12 106 (1.07)	2030 (1.07)	10 076 (1.07)	
Missing	36 563 (3.23)	6994 (3.70)	29 569 (3.14)	
Ethnicity^c^				
Hispanic or Latino	109 881 (9.70)	18 919 (10.00)	90 962 (9.65)	0.012
Not Hispanic or Latino	984 144 (86.92)	163 822 (86.62)	820 322 (86.98)	
Missing	38 195 (3.37)	6395 (3.38)	31 800 (3.37)	
Rurality				
Urban	792 915 (70.03)	133 484 (70.58)	659 431 (69.92)	0.014
Not urban or missing	339 305 (29.97)	55 652 (29.42)	283 653 (30.08)	
BMI, mean (SD)	31.30 (6.56)	31.39 (6.34)	31.29 (6.61)	0.016
Smoking status				
Never	447 672 (39.54)	75 114 (39.71)	372 558 (39.50)	0.017
Current	143 047 (12.63)	24 071 (12.73)	118 976 (12.62)	
Former	478 433 (42.26)	78 937 (41.74)	399 496 (42.36)	
Missing	63 068 (5.57)	11 014 (5.82)	52 054 (5.52)	
Nosos risk-adjustment score category (range)^d^				
1 (0-0.417)	28 804 (2.54)	5258 (2.78)	23 546 (2.50)	0.031
2 (0.417-0.471)	50 094 (4.42)	8823 (4.66)	41 271 (4.38)	
3 (0.471-0.534)	66 914 (5.91)	11 474 (6.07)	55 440 (5.88)	
4 (0.534-0.611)	82 930 (7.32)	13 933 (7.37)	68 997 (7.32)	
5 (0.611-0.707)	98 230 (8.68)	16 266 (8.60)	81 964 (8.69)	
6 (0.707-0.829)	115 034 (10.16)	18 828 (9.95)	96 206 (10.20)	
7 (0.829-0.998)	130 715 (11.55)	21 383 (11.31)	109 332 (11.59)	
8 (0.998-1.259)	148 904 (13.15)	24 384 (12.89)	124 520 (13.20)	
9 (1.259-1.805)	171 256 (15.13)	28 279 (14.95)	142 977 (15.16)	
10 (1.805-39.370)	212 683 (18.78)	36 243 (19.16)	176 440 (18.71)	
Missing	26 656 (2.35)	4265 (2.25)	22 391 (2.37)	
CAN score category (range)^e^				
1 (0-20)	187 321 (16.54)	32 459 (17.16)	154 862 (16.42)	0.030
2 (25-40)	175 137 (15.47)	29 541 (15.62)	145 596 (15.44)	
3 (45-60)	211 866 (18.71)	34 757 (18.38)	177 109 (18.78)	
4 (65-80)	256 709 (22.67)	42 021 (22.22)	214 688 (22.76)	
5 (85-90)	166 348 (14.69)	27 234 (14.40)	139 114 (14.75)	
6 (95-99)	112 499 (9.94)	19 526 (10.32)	92 973 (9.86)	
Missing	22 340 (1.97)	3598 (1.90)	18 742 (1.99)	
Gagne Index Score, mean (SD)^f^	1.40 (2.25)	1.40 (2.31)	1.40 (2.23)	<0.001
No. of VHA primary care contacts in previous 24 mo, mean (SD)	8.31 (10.27)	8.48 (9.59)	8.28 (10.40)	0.020
No. of VHA inpatient admissions in previous 24 mo, mean (SD)	0.35 (1.24)	0.35 (1.20)	0.35 (1.25)	0.001
No. of VHA specialty care visits in previous 24 mo, mean (SD)	13.50 (15.14)	13.82 (14.13)	13.44 (15.34)	0.026
COVID-19 vaccination before index date				
No	371 040 (32.77)	61 985 (32.77)	309 055 (32.77)	0.001
Yes	16 273 (1.44)	2743 (1.45)	13 530 (1.43)	
Vaccine not available	744 907 (65.79)	124 408 (65.78)	620 499 (65.79)	
CDC COVID-19 high-risk conditions				
Cancer	124 500 (11.00)	18 591 (9.83)	105 909 (11.23)	−0.046
Pulmonary	272 713 (24.09)	44 543 (23.55)	228 170 (24.19)	−0.015
Hypertension	725 547 (64.08)	119 550 (63.21)	605 997 (64.26)	−0.022
Diabetes	400 864 (35.41)	66 023 (34.91)	334 841 (35.50)	−0.013
Dementia	54 348 (4.80)	8870 (4.69)	45 478 (4.82)	−0.006
Coronary heart disease	318 599 (28.14)	52 448 (27.73)	266 151 (28.22)	−0.011
Sickle cell	2037 (0.18)	364 (0.19)	1673 (0.18)	0.004
Transplant	3704 (0.33)	599 (0.32)	3105 (0.33)	−0.002
Stroke or cerebrovascular disease	68 111 (6.02)	11 236 (5.94)	56 875 (6.03)	−0.004
Liver disease	118 322 (10.45)	19 726 (10.43)	98 596 (10.45)	−0.001
Kidney disease	252 183 (22.27)	41 951 (22.18)	210 232 (22.29)	−0.003
Congestive heart failure	115 236 (10.18)	19 239 (10.17)	95 997 (10.18)	<0.001
Major depression diagnosis	366 072 (32.33)	61 410 (32.47)	304 662 (32.30)	0.004
Anxiety diagnosis	256 722 (22.67)	43 236 (22.86)	213 486 (22.64)	0.005
PTSD diagnosis	289 301 (25.55)	48 514 (25.65)	240 787 (25.53)	0.003
Substance use disorder	141 515 (12.50)	23 845 (12.61)	117 670 (12.48)	0.004
Bipolar diagnosis	43 623 (3.85)	7322 (3.87)	36 301 (3.85)	−0.001
Schizophrenia diagnosis	24 985 (2.21)	4181 (2.21)	20 804 (2.21)	<0.001
Immunocompromised	110 335 (9.75)	18 437 (9.75)	91 898 (9.74)	<0.001
Community living center at index	10 426 (0.92)	1893 (1.00)	8533 (0.90)	0.010
Distance to nearest VAMC, mean (SD), m	35.64 (35.29)	35.12 (36.31)	35.75 (35.08)	−0.018
**Other variables (not used in matching)**					
Nonpreventable hospitalization count, 1-y post index, mean (SD)	0.30 (0.82)	0.50 (1.07)	0.26 (0.76)	0.264
COVID-19 pandemic wave of index diagnosis				
First (March-June 2020)	111 342 (9.83)	18 576 (9.82)	92 766 (9.84)	0.001
Second (July-Nov 2020)	397 985 (35.15)	66 478 (35.15)	331 507 (35.15)	
Third (Dec 2020-April 2021)	622 893 (55.02)	104 082 (55.03)	518 811 (55.01)	
Residence in primary care shortage area	272 998 (24.11)	47 147 (24.93)	225 851 (23.95)	0.023
Medicare Advantage at index	206 972 (18.28)	35 190 (18.61)	171 782 (18.21)	0.010
Long-term institutionalization during baseline or follow-up	51 634 (4.56)	11 507 (6.08)	40 127 (4.25)	0.083
Elixhauser rehospitalization risk score, mean (SD)	20.83 (22.73)	20.23 (22.92)	20.95 (22.69)	−0.032
Elixhauser rehospitalization risk score tertiles^g^				
Tertile 1 (−4 to 6)	377 407 (33.33)	65 444 (34.60)	311 963 (33.08)	0.045
Tertile 2 (6 to 24)	377 407 (33.33)	63 857 (33.76)	313 550 (33.25)	
Tertile 3 (24 to 187)	377 406 (33.33)	59 835 (31.64)	317 571 (33.67)	

Abbreviations: BMI, body mass index (calculated as weight in kilograms divided by height in meters squared); CAN, Care Assessment of Need; CDC, Centers for Disease Control and Prevention; PTSD, posttraumatic stress disorder; SMD, standardized mean difference; VAMC, Veterans Affair Medical Center; VHA, Veterans Health Administration.

^a^ Unless otherwise indicated, data are expressed as No. (%) of patients. Percentages have been rounded and may not total 100.

^b^ Pregnancy was a matching variable but was zero for all persons. State of residence was a matching variable and included 50 states and Washington, DC. Index month was an exact matching variable and spanned 14 months (not shown, absolute SMDs for both state of residence and index month <0.100).

^c^ Race and ethnicity data from the VHA electronic health record are collected through self-identification either at enrollment or at a health care encounter.

^d^ Scores are centered around 1. A value of 1 indicates that the veteran is expected to have annual health care costs that are the national average for VHA patients; a score higher than 1, the veteran has an expected annual health care cost that is higher than that of the average VHA patient; and a score lower than 1, the veteran has an expected annual health care cost that is lower than that of the average VHA patient. Overlapping score categories are mutually exclusive.

^e^ Higher scores indicate higher risk for hospitalization and/or mortality within the next 90 days.

^f^ Scores range from −2 to 22, with higher scores indicating higher comorbidity burden.

^g^ Higher scores indicate higher risk for rehospitalization within 1 year. Overlapping score categories are mutually exclusive.

### Potentially Preventable Hospitalizations

**Figure 1.**
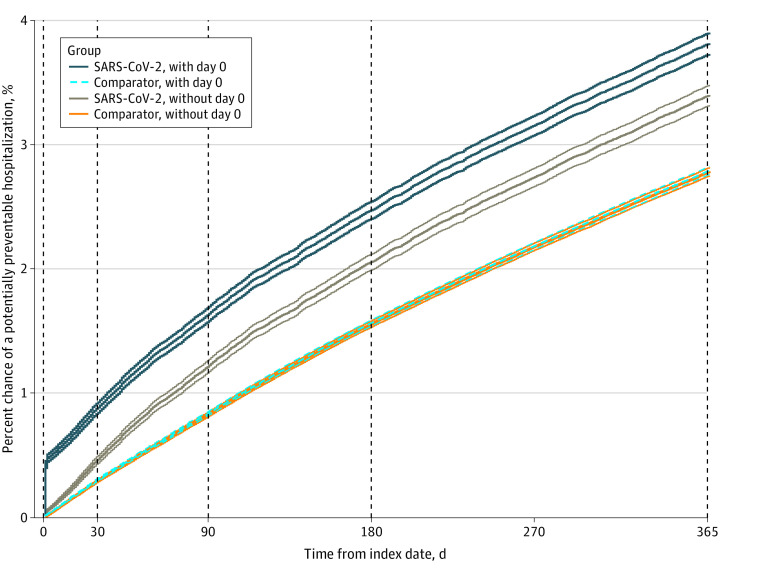
Cumulative Incidence of Potentially Preventable Hospitalizations During 1-Year Follow-Up Among Veterans With SARS-CoV-2 and Comparators Cumulative incidence curves are shown with 95% CIs. With day 0 includes potentially preventable hospitalizations on day 0 (index date), and without day 0 excludes these. There is substantial overlap in cumulative incidence and 95% CIs for comparators with and without day 0 hospitalizations. Cumulative incidence individually censors for death and crossover infection.

**Figure 2.**
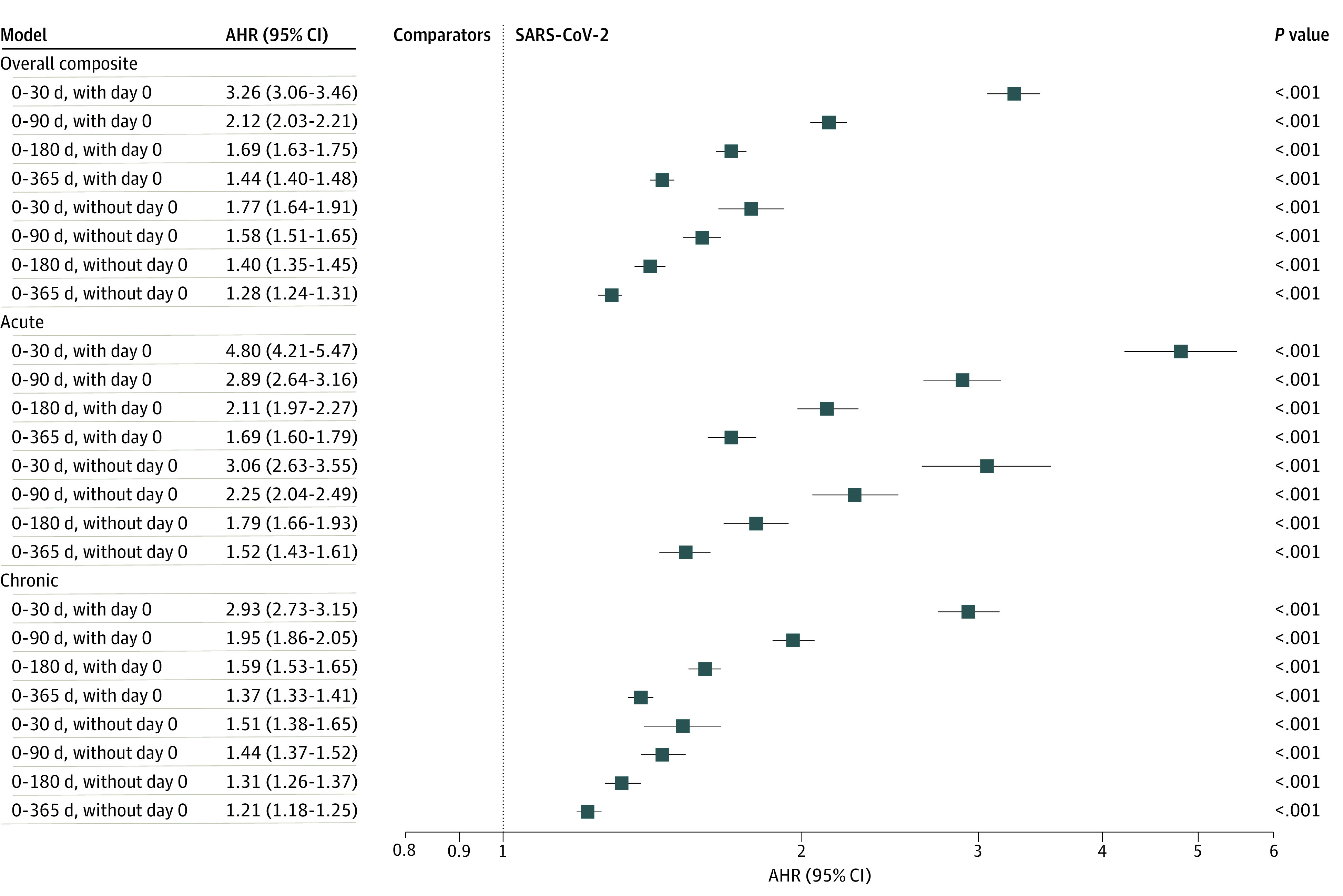
Adjusted Hazard Ratios (AHRs) of Potentially Preventable Hospitalization Among Veterans With SARS-CoV-2 Relative to Comparators Extended Cox models censor for deaths and crossover infections individually and are adjusted for nonpreventable hospitalizations as a time-varying covariate.

Overall, 3.10% of cohort members (3.81% of veterans with SARS-CoV-2 and 2.96% of comparators) had a potentially preventable hospitalization during 1-year follow-up (Table 2), with fewer than 1% of cohort members having more than 1. The cumulative incidence of potentially preventable hospitalizations grew more rapidly among veterans with SARS-CoV-2 than comparators during the first 90 days after the index date, with trends becoming similar with longer follow-up (Figure 1). The risk of a potentially preventable hospitalization was greater among veterans with SARS-CoV-2 than comparators during 4 cumulative follow-up periods, with the largest difference during the first 30 days after the index date (0.89% vs 0.31%; adjusted HR [AHR], 3.26 [95% CI, 3.06-3.46]) (Figure 2 and Table 2). Differences in risk between SARS-CoV-2 and comparator groups were smaller when assessed at 90 days (1.63% vs 0.86%; AHR, 2.12 [95% CI, 2.03-2.21]), 180 days (2.47% vs 1.63%; AHR, 1.69 [95% CI, 1.63-1.75]), and 365 days (3.81% vs 2.96%; AHR, 1.44 [95% CI, 1.40-1.48]). Sensitivity analyses, including when day 0 outcomes were excluded, yielded results similar in sign, magnitude, and significance (Figure 2, Table 2, and eTable 5 in Supplement 1).

**Table 2.** Incidence, Risk Difference, and Adjusted Hazards of Potentially Preventable Hospitalization Among Veterans With SARS-CoV-2 Relative to Comparators

Outcome	Incidence, No. (%)			Risk difference (95% CI), %	AHR (95% CI)^a^
	Overall (N = 1 132 220)	SARS-CoV-2 infection (n = 189 136)	Comparators (n = 943 084)		
**Primary analysis including day 0 hospitalizations**					
0-30 d					
Overall composite	4633 (0.41)	1686 (0.89)	2947 (0.31)	0.58 (0.54-0.62)	3.26 (3.06-3.46)
Acute	962 (0.09)	440 (0.23)	522 (0.06)	0.18 (0.16-0.20)	4.80 (4.21-5.47)
Chronic	3685 (0.33)	1254 (0.66)	2431 (0.26)	0.41 (0.37-0.44)	2.93 (2.73-3.15)
0-90 d					
Overall composite	11 185 (0.99)	3085 (1.63)	8100 (0.86)	0.77 (0.71-0.83)	2.12 (2.03-2.21)
Acute	2178 (0.19)	742 (0.39)	1436 (0.15)	0.24 (0.21-0.27)	2.89 (2.64-3.16)
Chronic	9028 (0.80)	2355 (1.25)	6673 (0.71)	0.54 (0.48-0.59)	1.95 (1.86-2.05)
0-180 d					
Overall composite	20 004 (1.77)	4669 (2.47)	15 335 (1.63)	0.84 (0.77-0.92)	1.69 (1.63-1.75)
Acute	3942 (0.35)	1083 (0.57)	2859 (0.30)	0.27 (0.23-0.31)	2.11 (1.97-2.27)
Chronic	16 099 (1.42)	3599 (1.90)	12 500 (1.33)	0.58 (0.51-0.64)	1.59 (1.53-1.65)
0-365 d					
Overall composite	35 146 (3.10)	7203 (3.81)	27 943 (2.96)	0.85 (0.75-0.94)	1.44 (1.40-1.48)
Acute	7246 (0.64)	1670 (0.88)	5576 (0.59)	0.29 (0.25-0.34)	1.69 (1.60-1.79)
Chronic	27 956 (2.47)	5549 (2.93)	22 407 (2.38)	0.56 (0.48-0.64)	1.37 (1.33-1.41)
**Primary analysis excluding day 0 hospitalizations**					
0-30 d					
Overall composite	3755 (0.33)	898 (0.47)	2857 (0.30)	0.17 (0.14-0.20)	1.77 (1.64-1.91)
Acute	775 (0.07)	272 (0.14)	503 (0.05)	0.09 (0.07-0.11)	3.06 (2.63-3.55)
Chronic	2994 (0.26)	634 (0.34)	2360 (0.25)	0.09 (0.06-0.11)	1.51 (1.38-1.65)
0-90 d					
Overall composite	10 307 (0.91)	2297 (1.21)	8010 (0.85)	0.37 (0.31-0.42)	1.58 (1.51-1.65)
Acute	1991 (0.18)	574 (0.30)	1417 (0.15)	0.15 (0.13-0.18)	2.25 (2.04-2.49)
Chronic	8337 (0.74)	1735 (0.92)	6602 (0.70)	0.22 (0.17-0.26)	1.44 (1.37-1.52)
0-180 d					
Overall composite	19 126 (1.69)	3881 (2.05)	15 245 (1.62)	0.44 (0.37-0.51)	1.40 (1.35-1.45)
Acute	3755 (0.33)	915 (0.48)	2840 (0.30)	0.18 (0.15-0.22)	1.79 (1.66-1.93)
Chronic	15 408 (1.36)	2979 (1.58)	12 429 (1.32)	0.26 (0.20-0.32)	1.31 (1.26-1.37)
0-365 d					
Overall composite	34 268 (3.03)	6415 (3.39)	27 853 (2.95)	0.44 (0.35-0.53)	1.28 (1.24-1.31)
Acute	7059 (0.62)	1502 (0.79)	5557 (0.59)	0.21 (0.16-0.25)	1.52 (1.43-1.61)
Chronic	27 265 (2.41)	4929 (2.61)	22 336 (2.37)	0.24 (0.16-0.32)	1.21 (1.18-1.25)

Abbreviation: AHR, adjusted hazard ratio.

^a^ Extended Cox regression models censor for deaths and crossover infections individually and are adjusted for nonpreventable hospitalizations as a time-varying covariate.

Potentially preventable hospitalizations for acute conditions (ie, bacterial pneumonia, urinary tract infection) accounted for 20.02% of all preventable hospitalizations; those for exacerbations of chronic conditions (eg, diabetes, asthma) accounted for the remainder (eTable 4 and eFigure 2 in Supplement 1 provide a breakout and differences by individual PQI). During the first 30 days after the index date, veterans with SARS-CoV-2 had greater risk than comparators of a potentially preventable hospitalization for acute conditions (0.23% vs 0.06%; AHR, 4.80 [95% CI, 4.21-5.47]) and exacerbations of chronic conditions (0.66% vs 0.26%; AHR, 2.93 [95% CI, 2.73-3.15]) (Figure 2 and Table 2). Although differences in risk became attenuated with longer follow-up, veterans with SARS-CoV-2 had greater risk than comparators of a potentially preventable hospitalization for acute and chronic conditions when assessed at 90 days (acute AHR, 2.89 [95% CI, 2.64-3.16]; chronic AHR, 1.95 [95% CI, 1.86-2.05]), 180 days (acute AHR, 2.11 [95% CI, 1.97-2.27]; chronic AHR, 1.59 [95% CI, 1.53-1.65]), and 365 days (acute AHR, 1.69 [95% CI, 1.60-1.79]; chronic AHR, 1.37 [95% CI, 1.33-1.41]).

### Exploratory Subgroup Results

**Figure 3.**
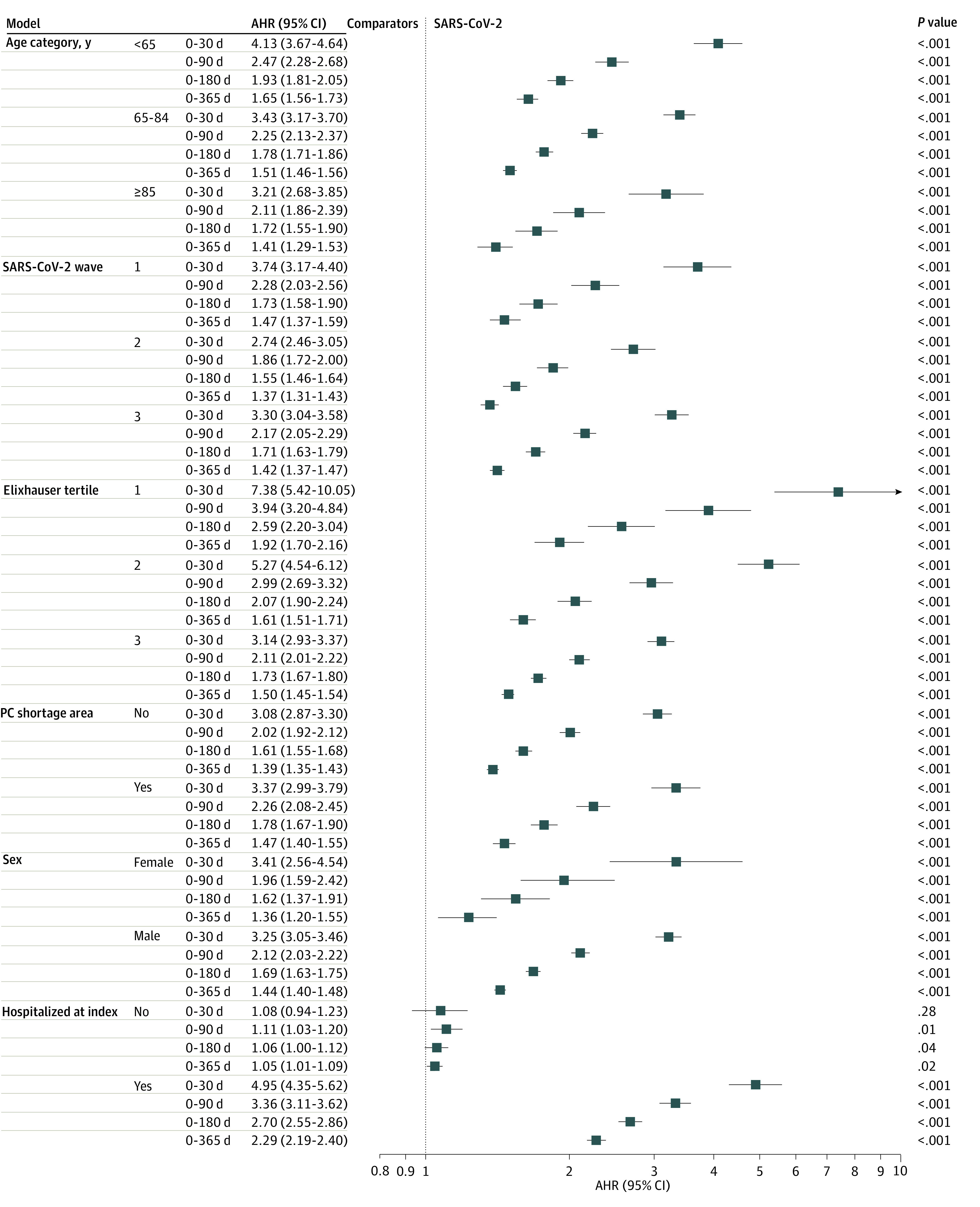
Subgroup Adjusted Hazard Ratios (AHRs) of Potentially Preventable Hospitalizations Among Veterans With SARS-CoV-2 Relative to Comparators Subgroup analyses censored for deaths and crossover infections individually. Elixhauser rehospitalization risk score tertile 1 indicates (−4 to 6); tertile 2, (6 to 24); and tertile 3, (24 to 187), where overlapping score categories are mutually exclusive. Primary care (PC) shortage area indicates veteran residence. All subgroup analyses controlled for nonoutcome hospitalizations. In addition, the following were controlled for in each subgroup analysis due to covariate imbalance within subgroups: for age subanalyses, Medicare Advantage enrollment at index, Care Assessment of Need score category, smoking status, Nosos category, and Gagne score; for COVID-19 wave subanalyses, no additional covariates; for Elixhauser rehospitalization risk score tertile subanalyses, VHA primary care visits in the 24 months prior to index date, VHA specialty care visits in the 24 months prior to index date, and Nosos category; for PC shortage area subanalyses, no additional covariates; and for sex subanalyses, no additional covariates.

For all subgroups we examined with 1 exception (0-30 day follow-up period for those not hospitalized at index date), risk of a potentially preventable hospitalization was greater among veterans with SARS-CoV-2 than among comparators in all follow-up periods, with some differences in the magnitudes of association (Figure 3 and eTable 6 in Supplement 1). For example, regarding pandemic waves, differences in risk between SARS-CoV-2 and comparator groups were larger during wave 1 (eg, 0- to 30-day AHR, 3.74 [95% CI, 3.17-4.40]) compared with wave 2 (eg, 0- to 30-day AHR, 2.74 [95% CI, 2.46-3.05]). Differences in risk between SARS-CoV-2 and comparator groups were also larger among those who resided in primary care shortage areas (eg, 0- to 30-day AHR, 3.37 [95% CI, 2.99-3.79]) compared with those not in a primary care shortage area (eg, 0- to 30-day AHR, 3.08 [95% CI, 2.87-3.30]). After excluding events that occurred on the same day as the index date, among those hospitalized at index, risk of a potentially preventable hospitalization was greater among veterans with SARS-CoV-2 than comparators (eg, 0- to 30-day AHR, 4.95 [95% CI, 4.35-5.62]). However, among those who were not hospitalized at the index date, differences in risk were not significant during the 0- to 30-day period. Veterans who were hospitalized at the index date had twice the overall incidence of potentially preventable hospitalization at 1-year follow-up than those who were not hospitalized at index (hospitalized: 4.69%, not hospitalized: 2.62%) (eTables 7-12 in Supplement 1 provide subgroup characteristics.)

## Discussion

In this cohort stuy using an emulated target trial design in the largest integrated health system of the US, approximately 1 in every 25 veterans with SARS-CoV-2 from March 1, 2020, to April 30, 2021 (3.81%), had a potentially preventable hospitalization in the year that followed, compared with approximately 1 in every 33 comparators (2.96%). Both groups had more hospitalizations for exacerbations of chronic conditions (eg, diabetes, asthma, heart failure) than for acute conditions (ie, bacterial pneumonia, urinary tract infection). During the 30-day follow-up, the risk of a potentially preventable hospitalization was more than 3 times higher among veterans with SARS-CoV-2 than comparators. Differences in risk became attenuated with longer follow-up time. However, when assessed through 1 year, veterans with SARS-Cov-2 had a 44% increased risk of a potentially preventable hospitalization relative to comparators. Expanding on prior studies reporting a positive association between SARS-CoV-2 and all-cause hospitalization,^14,15,17^ our results suggest that some postinfection hospitalizations need not occur.

Although results are exploratory, subgroup analyses suggest that suboptimal access to ambulatory care after infection increases the risk of a potentially preventable hospitalization among individuals with SARS-CoV-2. For example, the risk of a potentially preventable hospitalization was more pronounced for infections occurring during the earliest (March to June 2020) compared with latest (December 1, 2020, to April 30, 2021) pandemic wave, which represent a period of sweeping disruptions to care delivery,^9,10,11,12^ as well as in primary care professional shortage areas. However, additional research is needed to uncover causal mechanisms for these findings and to evaluate whether and for whom improved ambulatory care access may mitigate the risk of postinfection hospitalization. With a relaxing of telehealth regulations and increased payment parity during the COVID-19 pandemic,^32^ it will also be important to understand whether shifts from in-person to telehealth care have affected veterans’ risk of preventable hospitalization after SARS-CoV-2. In a recent analysis composed of a cohort similar to the one from our overall study, Hebert et al^33^ found that compared with matched comparators, veterans with SARS-CoV-2 experienced marked increases in outpatient visits during the first 30 days after infection, and that half of the additional outpatient visits were delivered via telehealth. Taken together, findings may suggest the importance of in-person care after SARS-CoV-2 for preventing adverse events such as hospitalization. However, more study is needed.

Overall, our findings suggest that care disruptions, which may have originated from individual and systems-level responses to the pandemic, may have induced consequential gaps in the treatment of ACSCs among individuals with SARS-CoV-2, leading to potentially preventable hospitalizations. Alternatively, in some cases, potentially preventable hospitalization may not be simply a proxy measure for ambulatory care access and quality, but rather may represent sudden disruptions in health care that can quickly lead to exacerbations among those with SARS-CoV-2. Research is needed to directly test the role of access to and quality of ambulatory care on postinfection outcomes as well as trade-offs between strategies that limit infection spread vs those that promote access to needed care during public health emergencies. Overall, more work is needed to understand how SARS-CoV-2 shapes postinfection care needs and interactions with the health system. With this information, care processes can be reconfigured to address these needs. Potentially preventable hospitalizations represent suboptimal use of limited health care resources; therefore, the economic effects of these events among veterans with SARS-CoV-2 should also be assessed.

### Strengths and Limitations

This study has several strengths. First, we assessed not only VHA-direct hospitalizations, but also VHA-purchased and Medicare fee-for-service hospitalizations. Second, we used the VA CSDR to identify SARS-CoV-2 infections.^18^ The CSDR includes VHA health care facility–, non-VHA health care facility–, and community-detected SARS-CoV-2 infections, affording us a high level of confidence in our ascertainment of veterans’ SARS-CoV-2 status. In addition, we used national electronic health record and administrative data to build cohorts composed of the universe of VHA-engaged veterans with SARS-CoV-2 infections and matched comparators. These data provided detailed information on the characteristics of our sample, enabling this detailed assessment of the association between SARS-CoV-2 and potentially preventable hospitalization.

We also acknowledge limitations to our study. First, we did not use Medicare Advantage, Medicaid, or commercial insurance data. Our results may therefore be biased if SARS-CoV-2 and comparator groups had differential patterns of these hospitalizations. However, analyses excluding veterans with Medicare Advantage yielded similar results, enhancing confidence in our findings. Second, untested or unreported SARS-CoV-2 infections occurred with greater frequency as the pandemic progressed, which could bias our results toward the null. Third, we used a directed acyclic graph–informed design to maximize rigor in this observational study.^19^ However, as in all observational studies, we cannot rule out bias due to unmeasured confounding. Last, our results may not generalize beyond the VHA-engaged veteran population, which has different characteristics (eg, older age, greater disability) than the general US population.

## Conclusions

In this cohort study, the risk of a potentially preventable hospitalization—a measure of ambulatory care access and quality as well as health system response to public health emergencies—was greater among veterans with SARS-CoV-2 than among matched comparators without SARS-CoV-2. Differences in risk between groups were largest during 30-day follow-up and became attenuated with longer follow-up. However, risk of potentially preventable hospitalization assessed at 1 year was more than 40% greater among veterans with SARS-CoV-2. Our findings highlight the need for research on the ways in which SARS-CoV-2 shapes postinfection health care needs and interactions with the health system.

## Notes

Supplement 1. **eTable 1.** Emulated Target Trial Approach **eTable 2.** Variables Used in Exact and Propensity Score Matching **eFigure 1.** Study Flow Diagram **eTable 3.** AHRQ Prevention Quality Indicators (PQIs) for Potentially Preventable Hospitalizations **eTable 4.** Potentially Preventable Hospitalizations Among Veterans With SARS-CoV-2 and Matched Comparators During 4 Cumulative Follow-Up Periods, Individual Prevention Quality Indicators (PQIs) **eFigure 2.** Differences in Percentages (95% CI) of Potentially Preventable Hospitalizations Between Veterans With SARS-CoV-2 and Matched Uninfected Comparators During 1-Year Follow-Up, Individual Prevention Quality Indicators (PQIs) **eTable 5.** Sensitivity Analysis Results (Adjusted Hazard Ratio [95% CI]) for Overall Composite Potentially Preventable Hospitalization Among Veterans With SARS-CoV-2 Relative to Matched Comparators **eTable 6.** Subgroup Incidence, Risk Difference, and Adjusted Hazards (95% CI) for Overall Composite Potentially Preventable Hospitalization Among Veterans With SARS-CoV-2 and Matched Comparators **eTable 7.** Sample Characteristics for Veterans With SARS-CoV-2 and Matched Comparators by Age Group **eTable 8.** Sample Characteristics for Veterans With SARS-CoV-2 and Matched Comparators by Sex **eTable 9.** Sample Characteristics for Veterans With SARS-CoV-2 and Matched Comparators by Elixhauser Rehospitalization Score Tertile **eTable 10.** Sample Characteristics for Veterans With SARS-CoV-2 and Matched Comparators by Residence in a Primary Care Shortage Area **eTable 11.** Sample Characteristics for Veterans With SARS-CoV-2 and Matched Uninfected Comparators by COVID-19 Pandemic Wave of Index Date (ie, Infection Date) **eTable 12.** Sample Characteristics for Veterans With SARS-CoV-2 and Matched Comparators by Hospitalization at Index

Supplement 2.
Nonauthor Collaborators

Supplement 3.
Data Sharing Statement
