# Supplementary material for: Risk of Potentially Preventable Hospitalizations After SARS-CoV-2 Infection
Source: JAMA Netw Open. 2024 Apr 10;7(4):e245786. doi: 10.1001/jamanetworkopen.2024.5786 (PMC11007577; doi:10.1001/jamanetworkopen.2024.5786)
Supplement: Supplement 1. — eTable 1. Emulated Target Trial Approach eTable 2. Variables Used in Exact and Propensity Score Matching eFigure 1. Study Flow Diagram eTable 3. AHRQ Prevention Quality Indicators (PQIs) for Potentially Preventable Hospitalizations eTable 4. Potentially Preventable Hospitalizations Among Veterans With SARS-CoV-2 and Matched Comparators During 4 Cumulative Follow-Up Periods, Individual Prevention Quality Indicators (PQIs) eFigure 2. Differences in Percentages (95% CI) of Potentially Preventable Hospitalizations Between Veterans With SARS-CoV-2 and Matched Uninfected Comparators During 1-Year Follow-Up, Individual Prevention Quality Indicators (PQIs) eTable 5. Sensitivity Analysis Results (Adjusted Hazard Ratio [95% CI]) for Overall Composite Potentially Preventable Hospitalization Among Veterans With SARS-CoV-2 Relative to Matched Comparators eTable 6. Subgroup Incidence, Risk Difference, and Adjusted Hazards (95% CI) for Overall Composite Potentially Preventable Hospitalization Among Veterans With SARS-CoV-2 and Matched Comparators eTable 7. Sample Characteristics for Veterans With SARS-CoV-2 and Matched Comparators by Age Group eTable 8. Sample Characteristics for Veterans With SARS-CoV-2 and Matched Comparators by Sex eTable 9. Sample Characteristics for Veterans With SARS-CoV-2 and Matched Comparators by Elixhauser Rehospitalization Score Tertile eTable 10. Sample Characteristics for Veterans With SARS-CoV-2 and Matched Comparators by Residence in a Primary Care Shortage Area eTable 11. Sample Characteristics for Veterans With SARS-CoV-2 and Matched Uninfected Comparators by COVID-19 Pandemic Wave of Index Date (ie, Infection Date) eTable 12. Sample Characteristics for Veterans With SARS-CoV-2 and Matched Comparators by Hospitalization at Index [file jamanetwopen-e245786-s001.pdf]

Supplementary Online Content

Govier DJ, Niederhausen M, Takata Y, et al; US Department of Veterans Affairs Health Services Research and Development COVID-19 Observational Research Collaboratory. Risk of potentially preventable hospitalizations after SARS-CoV-2 infection. *JAMA Netw Open*. 2024;7(4):e245786. doi:10.1001/jamanetworkopen.2024.5786

eTable 1. Emulated Target Trial Approach
eTable 2. Variables Used in Exact and Propensity Score Matching
eFigure 1. Study Flow Diagram
eTable 3. AHRQ Prevention Quality Indicators (PQIs) for Potentially Preventable Hospitalizations
eTable 4. Potentially Preventable Hospitalizations Among Veterans With SARS-CoV-2 and Matched Comparators During 4 Cumulative Follow-Up Periods, Individual Prevention Quality Indicators (PQIs)
eFigure 2. Differences in Percentages (95% CI) of Potentially Preventable Hospitalizations Between Veterans With SARS-CoV-2 and Matched Uninfected Comparators During 1-Year Follow-Up, Individual Prevention Quality Indicators (PQIs)
eTable 5. Sensitivity Analysis Results (Adjusted Hazard Ratio [95% CI]) for Overall Composite Potentially Preventable Hospitalization Among Veterans With SARS-CoV-2 Relative to Matched Comparators
eTable 6. Subgroup Incidence, Risk Difference, and Adjusted Hazards (95% CI) for Overall Composite Potentially Preventable Hospitalization Among Veterans With SARS-CoV-2 and Matched Comparators
eTable 7. Sample Characteristics for Veterans With SARS-CoV-2 and Matched Comparators, by Age Group
eTable 8. Sample Characteristics for Veterans With SARS-CoV-2 and Matched Comparators, by Sex
eTable 9. Sample Characteristics for Veterans With SARS-CoV-2 and Matched Comparators, by Elixhauser Rehospitalization Score Tertile
eTable 10. Sample Characteristics for Veterans With SARS-CoV-2 and Matched Comparators, by Residence in a Primary Care Shortage Area
eTable 11. Sample Characteristics for Veterans With SARS-CoV-2 and Matched Uninfected Comparators, by COVID-19 Pandemic Wave of Index Date (ie, Infection Date)
eTable 12. Sample Characteristics for Veterans With SARS-CoV-2 and Matched Comparators, by Hospitalization at Index

This supplementary material has been provided by the authors to give readers additional information about their work.

© 2024 Govier DJ et al. *JAMA Network Open*.

eTable 1. Emulated Target Trial Approach

	Unethical Target Trial	Emulation
**Goal**	To test the effect of individual infection with SARS-CoV-2 on potentially preventable hospitalization at 30, 90, 180, and 365 days after infection	Same
**Setting**	VHA nationwide system	Same
**Inclusion Criteria**	Veterans aged 18 and above in care in the VHA with an assigned primary care team for at least two years on randomization date, or who had at least one VHA primary care clinic visit in that period	Same
**Exclusion Criteria**	Previous SARS-CoV-2 Infection<br><br>Address outside of DC or 50 States	Previous documented SARS-CoV-2 Infection in National Surveillance tool or Medicare-documented SARS-CoV-2 diagnosis or related diagnostic codes (ICD-10: B97.29, U07.1, U09.9, J12.82, 179 Z86.16) listed in fee-for-service Medicare claims<br>Address outside of DC or 50 States<br>Missing or invalid key matching variables: age, height, weight, ZIP code<br>No suitable matches between infected patients and comparator
**Enrollment Period**	October 2020—April, 2021	Same
**"Treatment" Strategies**	Inoculum of SARS-CoV-2 sufficient to guarantee SARS-CoV-2 infection	SARS-CoV-2 Infection with a confirmatory PCR test for SARS-CoV-2 in VHA National Surveillance Tool
**Comparator**	Double-blinded inoculum of placebo	Best matched Veteran with neither documented SARS-CoV-2 Infection in National Surveillance tool nor Medicare-documented SARS-CoV-2 diagnosis through the month at which matched as a comparator
**Approach to balancing confounders**	1:1 Randomization, stratified by month and center	Up to 5:1 (Comparator-to-Infected) Matching on 5 exact criteria (including month and home state) and 37 propensity score criteria from VHA data
**Primary Outcome**	Composite Prevention Quality Indicators (PQIs) for potentially preventable hospitalizations of any kind, of the acute kind, and of the chronic kind	Same
**Follow-up Period**	30, 90, 180, and 365 days from inoculation	30, 90, 180, and 365 days from the earliest date of a documented positive test for those with SARS-CoV-2 infection; comparators

© 2024 Govier DJ et al. *JAMA Network Open*.

	Unethical Target Trial	Emulation
		began surveillance for outcomes from the same date (“index date”, the emulated equivalent of “randomization and inoculation date”) as that of their individually matched infected patient and were also followed for 30, 90, 180, and 365 days
**Causal Contrast**	Primary Analysis: comparison of outcomes between survivors of SARS-CoV-2 and those without contemporaneous infection	Primary Analysis: same

eTable 1 Footnotes: Abbreviations: VHA Veterans Health Administration; SARS-CoV-2 severe acute respiratory syndrome coronavirus version 2; PCR polymerase chain reaction; ICD-10 International classification of diseases tenth revision clinical modification.

eTable 2. Variables Used in Exact and Propensity Score Matching

Exact-Matched Variables	Categorical Propensity Score-Matched Variables	Continuous Propensity Score-Matched Variables
Sex, immunosuppressive medication use, state of residence (and Washington DC), and vaccination status (January-April 2021)	Sex, immunosuppressive medication use, vaccination status (January-April 2021), nursing home residence, race, ethnicity, rurality, smoking status, Can score, Nosos Risk Adjustment score, and indicators for diagnosed CDC high-risk conditions based on ICD-19 codes: coronary heart disease, cancer (excluding non-metastatic skin cancers), chronic kidney disease, congestive heart failure, pulmonary-associated conditions (including asthma, COPD, interstitial lung disease, and cystic fibrosis), dementia, diabetes, hypertension, liver disease, sickle cell/thalassemia, solid organ or blood stem cell transplant, stroke/cerebrovascular disorders, substance use disorder, anxiety disorder, bipolar disorder, major depression, PTSD, and schizophrenia	Age, body mass index, comorbidity score via Gagne index, number VHA inpatient admissions in two years prior to index date, number VHA primary care visits in two years prior to index date, number VHA specialty care visits in two years prior to index date, and number VHA mental health visits in two years prior to index date, and distance to nearest VHA medical center

eTable2 Footnotes: Race and ethnicity data from the VA electronic health record are collected through self-identification either at enrollment or at a health care encounter.

© 2024 Govier DJ et al. *JAMA Network Open*.

eFigure 1. Study Flow Diagram

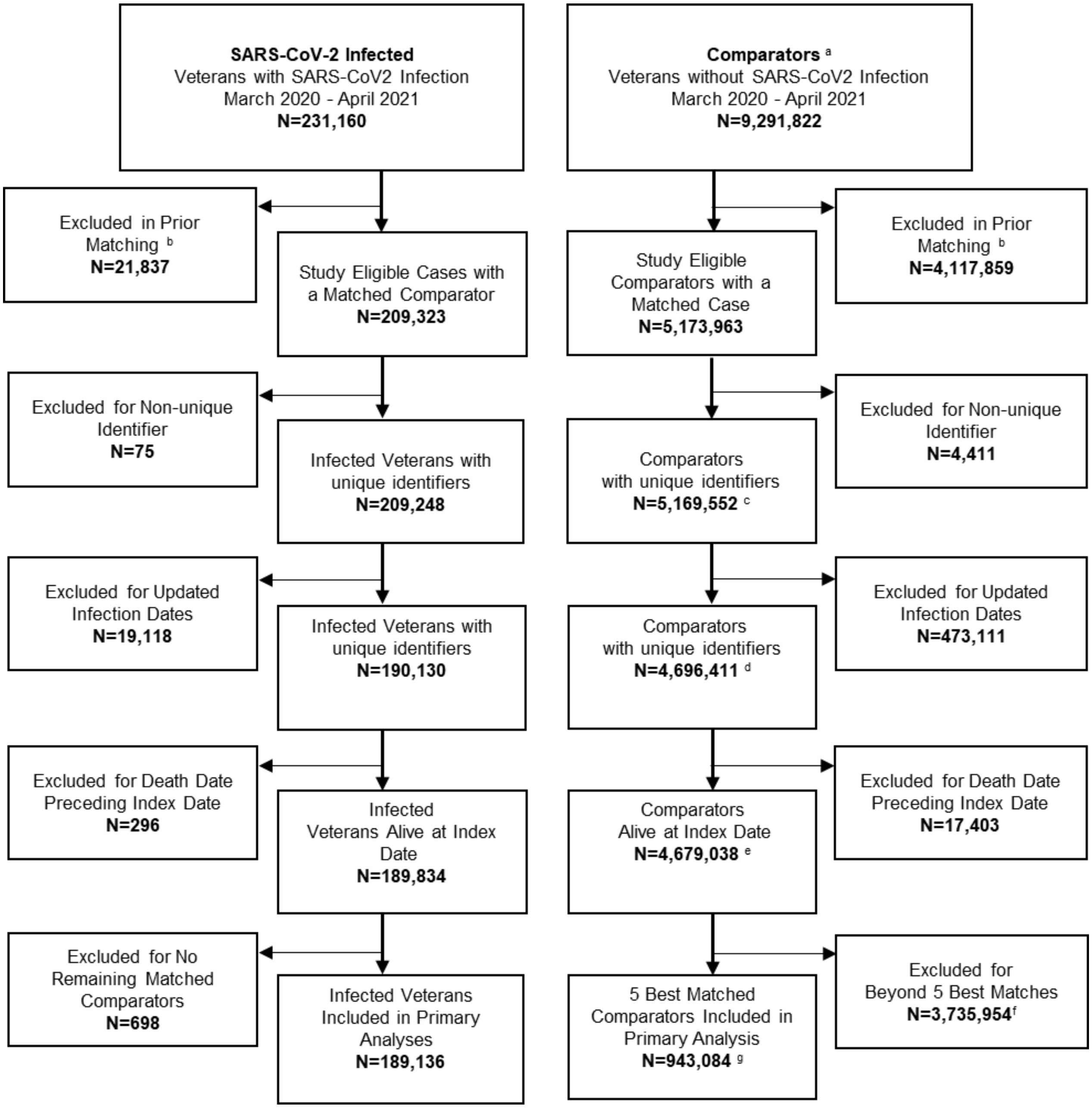

[a] Comparators that were matched to more than one infected Veteran have been counted as many times as they were matched. Numbers of unique comparators are presented in the footnotes below.
[b] Observations excluded prior to matching include those with missing CAN score; no enrollment in VHA Patient Aligned Care Team or primary care encounters; missing or implausible height, weight, age; missing Zip code or not in District of Columbia or 50 U.S. states; documented SARS-CoV-2 infection in Medicare data prior to VHA documented infection; no suitable match. Comparators were also excluded if they became infected during index month.
[c] Unique number of uninfected comparators matched to a Veteran with SARS-CoV-2: N=3,012,579
[d] Updated testing information was used to improve ascertainment of index dates. Comparators were excluded if the infection date of their matched Veteran with SARS-CoV-2 was determined inaccurate based on updated data. Unique number of matched comparators with unique identifiers: N=2,845,864
[e] Unique number of alive matched comparators at index date: N=2,837,492
[f] Unique number of matched comparators that were dropped: N=2,465,030
[g] Unique number of matched comparators used for analysis: N=832,057. Of the comparators included in the analysis, 95,220 were matched to more than one Veteran with SARS-CoV-2.

© 2024 Govier DJ et al. *JAMA Network Open*.

eTable 3. AHRQ Prevention Quality Indicators (PQIs) for Potentially Preventable Hospitalizations

**PQI 90 Prevention Quality Overall Composite**
PQI 91 Prevention Quality Acute Composite
PQI 92 Prevention Quality Chronic Composite
**PQI 91 Prevention Quality Acute Composite**
PQI 11 Bacterial Pneumonia Admission Rate
PQI 12 Urinary Tract Infection Admission Rate
**PQI 92 Prevention Quality Chronic Composite**
PQI 01 Diabetes Short-Term Complications Admission Rate
PQI 03 Diabetes Long-Term Complications Admission Rate
PQI 05 Chronic Obstructive Pulmonary Disease (COPD) or Asthma in Older Adults Admission Rate
PQI 07 Hypertension Admission Rate
PQI 08 Heart Failure Admission Rate
PQI 14 Uncontrolled Diabetes Admission Rate
PQI 15 Asthma in Younger Adults Admission Rate
PQI 16 Lower-Extremity Amputation among Patients with Diabetes Rate

© 2024 Govier DJ et al. *JAMA Network Open*.

eTable 4. Potentially Preventable Hospitalizations Among Veterans With SARS-CoV-2 and Matched Comparators During 4 Cumulative Follow-Up Periods, Individual Prevention Quality Indicators (PQIs)

	Days 0-30	Days 0-90	Days 0-180	Days 0-365
**PQI 91 Prevention Quality Acute Composite**				
PQI 11 – Community-Acquired Pneumonia				
Overall, n (%)	597 (0.05)	1,277 (0.11)	2,246 (0.20)	4,096 (0.36)
SARS-CoV-2, n (%)	284 (0.15)	465 (0.25)	625 (0.33)	928 (0.49)
Comparators, n (%)	313 (0.03)	812 (0.09)	1,621 (0.17)	3,168 (0.34)
Unadjusted Risk Difference, %-point (95% CI)	0.12 (0.10, 0.14)	0.16 (0.14, 0.18)	0.16 (0.13, 0.19)	0.16 (0.12, 0.19)
PQI 12 – Urinary Tract Infection				
Overall, n (%)	365 (0.03)	902 (0.08)	1,697 (0.15)	3,151 (0.28)
SARS-CoV-2, n (%)	156 (0.08)	278 (0.15)	459 (0.24)	743 (0.39)
Comparators, n (%)	209 (0.02)	624 (0.07)	1,238 (0.13)	2,408 (0.26)
Unadjusted Risk Difference, %-point (95% CI)	0.06 (0.05, 0.07)	0.08 (0.06, 0.10)	0.11 (0.09, 0.14)	0.14 (0.11, 0.17)
**PQI 92 Prevention Quality Chronic Composite**				
PQI 01 – Diabetes Short-Term				
Overall, n (%)	255 (0.02)	508 (0.05)	878 (0.08)	1,490 (0.13)
SARS-CoV-2, n (%)	139 (0.07)	191 (0.10)	249 (0.13)	353 (0.19)
Comparators, n (%)	116 (0.01)	317 (0.03)	629 (0.07)	1,137 (0.12)
Unadjusted Risk Difference, %-point (95% CI)	0.06 (0.05, 0.07)	0.07 (0.05, 0.08)	0.07 (0.05, 0.08)	0.07 (0.05, 0.09)
PQI 03 – Diabetes Long-Term				
Overall, n (%)	461 (0.04)	1,150 (0.10)	2,099 (0.19)	3,761 (0.33)
SARS-CoV-2, n (%)	158 (0.08)	292 (0.15)	472 (0.25)	744 (0.39)
Comparators, n (%)	303 (0.03)	858 (0.09)	1,627 (0.17)	3,017 (0.32)
Unadjusted Risk Difference, %-point (95% CI)	0.05 (0.04, 0.07)	0.06 (0.04, 0.08)	0.08 (0.05, 0.10)	0.07 (0.04, 0.10)
PQI 05 – COPD/Asthma Older Adults				
Overall, n (%)	515 (0.05)	1,284 (0.11)	2,470 (0.22)	4,447 (0.39)
SARS-CoV-2, n (%)	168 (0.09)	312 (0.17)	527 (0.28)	904 (0.48)
Comparators, n (%)	347 (0.04)	972 (0.10)	1,943 (0.21)	3,543 (0.38)
Unadjusted Risk Difference, %-point (95% CI)	0.05 (0.04, 0.07)	0.06 (0.04, 0.08)	0.07 (0.05, 0.10)	0.10 (0.07, 0.14)
PQI 07 - Hypertension				
Overall, n (%)	311 (0.03)	731 (0.07)	1,313 (0.12)	2,345 (0.21)
SARS-CoV-2, n (%)	110 (0.06)	191 (0.10)	303 (0.16)	482 (0.26)
Comparators, n (%)	201 (0.02)	540 (0.06)	1,010 (0.11)	1,863 (0.20)
Unadjusted Risk Difference, %-point (95% CI)	0.04 (0.03, 0.05)	0.04 (0.03, 0.06)	0.05 (0.03, 0.07)	0.06 (0.03, 0.08)
PQI 08 – Heat Failure				
Overall, n (%)	1,786 (0.16)	4,594 (0.41)	8,048 (0.71)	13,659 (1.21)

© 2024 Govier DJ et al. *JAMA Network Open*.

	Days 0-30	Days 0-90	Days 0-180	Days 0-365
SARS-CoV-2, n (%)	504 (0.27)	1,105 (0.58)	1,695 (0.90)	2,532 (1.34)
Comparators, n (%)	1,282 (0.14)	3,489 (0.37)	6,353 (0.67)	11,127 (1.18)
Unadjusted Risk Difference, %-point (95% CI)	0.13 (0.11, 0.16)	0.21 (0.18, 0.25)	0.22 (0.18, 0.27)	0.16 (0.10, 0.22)
PQI 14 – Uncontrolled Diabetes				
Overall, n (%)	286 (0.03)	584 (0.05)	996 (0.09)	1,744 (0.15)
SARS-CoV-2, n (%)	146 (0.08)	214 (0.11)	283 (0.15)	425 (0.23)
Comparators, n (%)	140 (0.02)	370 (0.04)	713 (0.08)	1,319 (0.14)
Unadjusted Risk Difference, %-point (95% CI)	0.06 (0.05, 0.08)	0.07 (0.06, 0.09)	0.07 (0.06, 0.09)	0.09 (0.06, 0.11)
PQI 15 – Asthma, Younger Adults				
Overall, n (%)	42 (<0.01)	91 (0.01)	167 (0.02)	300 (0.03)
SARS-CoV-2, n (%)	13 (0.01)	25 (0.01)	39 (0.02)	68 (0.04)
Comparators, N (%)	29 (<0.01)	66 (0.01)	128 (0.01)	232 (0.03)
Unadjusted Risk Difference, %-point (95% CI)	0.004 (<0.001, 0.01)	0.01 (<0.001, 0.01)	0.01(<0.001, 0.01)	0.01 (0.002, 0.02)
PQI 16 – Lower Extremity Amputation Among Patients with Diabetes				
Overall, n (%)	193 (0.02)	454 (0.04)	734 (0.07)	1,204 (0.11)
SARS-CoV-2, n (%)	79 (0.04)	141 (0.08)	183 (0.10)	258 (0.14)
Comparators, n (%)	114 (0.01)	313 (0.03)	551 (0.06)	946 (0.10)
Unadjusted Risk Difference, %-point (95% CI)	0.03 (0.02, 0.04)	0.04 (0.03, 0.06)	0.04 (0.02, 0.05)	0.04 (0.02, 0.05)

eTable 4 Footnotes: Abbreviations: PQI: prevention quality indicator; CI: confidence interval

© 2024 Govier DJ et al. *JAMA Network Open*.

eFigure 2. Differences in Percentages (95% CI) of Potentially Preventable Hospitalizations Between Veterans With SARS-CoV-2 and Matched Uninfected Comparators During 1-Year Follow-Up, Individual Prevention Quality Indicators (PQIs)

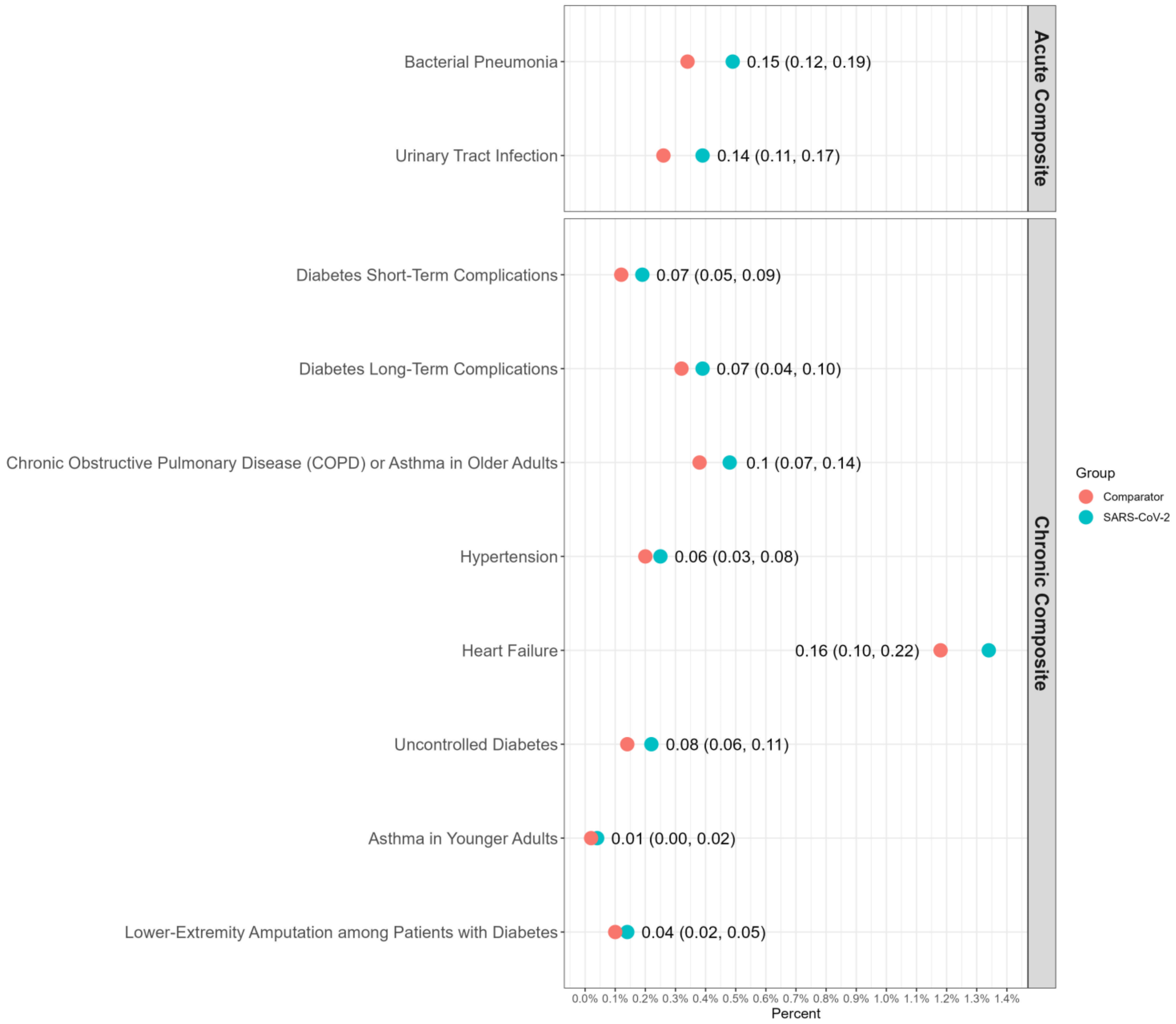

eFigure 2 Footnotes: Abbreviations: PQI: prevention quality indicator; CI: confidence interval

© 2024 Govier DJ et al. *JAMA Network Open*.

eTable 5. Sensitivity Analysis Results (Adjusted Hazard Ratio [95% CI]) for Overall Composite Potentially Preventable Hospitalization Among Veterans With SARS-CoV-2 Relative to Matched Comparators

A

Follow-up Period (Days)	Primary Analysis (PP1)[a]	Excluding Day-Zero Outcomes[b]	Excluding Medicare Advantage[c]	Excluding Institutionalized[d]	Per-Protocol 2 (PP2)[e]
0-30	3.26 (3.06, 3.46)	1.77 (1.64, 1.91)	2.95 (2.74, 3.18)	3.47 (3.23, 3.74)	2.28 (2.13, 2.45)
0-90	2.12 (2.03, 2.21)	1.58 (1.51, 1.65)	1.93 (1.83, 2.03)	2.21 (2.10, 2.33)	1.77 (1.69, 1.85)
0-180	1.69 (1.63, 1.75)	1.40 (1.35, 1.45)	1.54 (1.48, 1.61)	1.70 (1.64, 1.77)	1.51 (1.46, 1.57)
0-365	1.44 (1.40, 1.48)	1.28 (1.24, 1.31)	1.33 (1.29, 1.37)	1.43 (1.39, 1.48)	1.35 (1.31, 1.39)

eTable 5 Footnotes: PP1: Per-Protocol 1, PP2: Per-Protocol 2; CI: confidence interval

[a] Primary analysis (PP1): Extended Cox models with a baseline stratified hazard specification based on matched group. Models censor for death individually and crossover infections among comparators individually and adjusted for non-preventable hospitalizations as a time-varying covariate. Overall N=1,132,220; SARS-CoV-2 n=189,136, Comparator n=943,084.

[b] Same as PP1 except potentially preventable hospitalizations that occurred on the same day as index date were excluded from analysis. Overall N=1,132,220; SARS-CoV-2 n=189,136, Comparator n=943,084.

[c] Same as PP1, except individual comparators with Medicare Advantage coverage at baseline and matched groups in which the Veteran with SARS-CoV-2 had Medicare Advantage at baseline were excluded. Overall N=785,232; SARS-CoV-2 n=153,826, Comparator n=631,406.

[d] Same as PP1, except individual comparators who were institutionalized at baseline or follow-up, and matched groups in which the Veteran with SARS-CoV-2 was institutionalized at baseline or follow-up were excluded. Institutionalization defined as any inpatient stay lasting >180 days. Overall N=921,452; SARS-CoV-2 n=153,946, Comparator n=767,506.

[e] Per-Protocol-2 (PP2): Extended Cox models with a baseline stratified hazard specification based on matched group. Models censor for deaths individually and crossover infections among comparators by matched group (vs. individually) and adjusted for non-preventable hospitalizations as a time-varying covariate. Overall N=1,132,220; SARS-CoV-2 n=189,136, Comparator n=943,084.

B

Follow-up Period (Days)	Primary Analysis (PP1)[a]	Follow-up Period (Days)	Discrete Follow-up Intervals[b]
0-30	3.26 (3.06, 3.46)	0-30	3.26 (3.06, 3.46)
0-90	2.12 (2.03, 2.21)	31-90	1.48 (1.40, 1.58)
0-180	1.69 (1.63, 1.75)	91-180	1.21 (1.14, 1.28)
0-365	1.44 (1.40, 1.48)	181-365	1.13 (1.08, 1.18)

eTable 5 Footnotes: PP1: Per-Protocol 1; CI: confidence interval

[a] Primary analysis (PP1): Extended Cox models with a baseline stratified hazard specification based on matched group, with cumulative follow-up periods of 0-30 days, 0-90 days, 0-181 days, and 0-365 days. Models censor for death individually and crossover infections among comparators individually and adjusted for non-preventable hospitalizations as a time-varying covariate. Overall N=1,132,220; SARS-CoV-2 n=189,136, Comparator n=943,084.

[b] Extended Cox models with a baseline stratified hazard specification based on matched group, with discrete follow-up intervals. Individuals no longer at risk for the outcome due to events in prior time intervals are censored. Models additionally censor for death individually and crossover infections among comparators individually and adjusted for non-preventable hospitalizations as a time-varying covariate. Overall N=1,132,220; SARS-CoV-2 n=189,136, Comparator n=943,084.

eTable 6. Subgroup Incidence, Risk Difference, and Adjusted Hazards (95% CI) for Overall Composite Potentially Preventable Hospitalization Among Veterans With SARS-CoV-2 and Matched Comparators

	Overall Incidence, n (%)	Incidence in SARS-CoV-2, n (%)	Incidence in Comparators, n (%)	Risk Difference, % (95% CI)	Adjusted Hazard Ratio (95% CI)
**Sex**					
Male					
Day 0-30	4,434 (0.44)	1,609 (0.96)	2,825 (0.34)	0.62 (0.57, 0.67)	3.25 (3.05, 3.46)
Day 0-90	10,742 (1.07)	2,963 (1.76)	7,779 (0.93)	0.83 (0.77, 0.90)	2.12 (2.03, 2.22)
Day 0-180	19,211 (1.91)	4,479 (2.66)	14,732 (1.75)	0.91 (0.82, 0.99)	1.69 (1.63, 1.75)
Day 0-365	33,724 (3.34)	6,908 (4.10)	26,816 (3.19)	0.91 (0.81, 1.01)	1.44 (1.40, 1.48)
Female					
Day 0-30	199 (0.16)	77 (0.37)	122 (0.12)	0.25 (0.17, 0.34)	3.41 (2.56, 4.54)
Day 0-90	443 (0.36)	122 (0.59)	321 (0.31)	0.28 (0.16, 0.39)	1.96 (1.59, 2.42)
Day 0-180	793 (0.64)	190 (0.92)	603 (0.59)	0.33 (0.19, 0.47)	1.62 (1.37, 1.91)
Day 0-365	1,422 (1.15)	295 (1.42)	1,127 (1.09)	0.33 (0.15, 0.51)	1.36 (1.20, 1.55)
**Age Group**					
<65					
Day 0-30	1,167 (0.19)	505 (0.47)	662 (0.13)	0.34 (0.29, 0.38)	4.13 (3.67, 4.64)
Day 0-90	2,721 (0.44)	872 (0.81)	1,849 (0.37)	0.44 (0.38, 0.50)	2.47 (2.28, 2.68)
Day 0-180	4,818 (0.78)	1,305 (1.21)	3,513 (0.69)	0.51 (0.44, 0.58)	1.93 (1.81, 2.05)
Day 0-365	8,485 (1.38)	2,033 (1.88)	6,452 (1.27)	0.61 (0.52, 0.69)	1.65 (1.56, 1.73)
65-84					
Day 0-30	2,905 (0.63)	1,006 (1.39)	1,899 (0.49)	0.91 (0.82, 1.00)	3.43 (3.17, 3.70)
Day 0-90	7,069 (1.53)	1,890 (2.62)	5,179 (1.33)	1.29 (1.17, 1.41)	2.25 (2.13, 2.37)
Day 0-180	12,705 (2.75)	2,877 (3.99)	9,828 (2.52)	1.47 (1.32, 1.62)	1.78 (1.71, 1.86)
Day 0-365	22,432 (4.85)	4,463 (6.18)	17,969 (4.60)	1.58 (1.39, 1.77)	1.51 (1.46, 1.56)
85+					
Day 0-30	561 (1.03)	175 (2.01)	386 (0.84)	1.17 (0.85, 1.48)	3.21 (2.68, 3.85)
Day 0-90	1,395 (2.56)	323 (3.71)	1,072 (2.34)	1.37 (0.94, 1.80)	2.11 (1.86, 2.39)
Day 0-180	2,481 (4.55)	487 (5.59)	1,994 (4.35)	1.24 (0.72, 1.77)	1.72 (1.55, 1.90)
Day 0-365	4,229 (7.75)	707 (8.12)	3,522 (7.68)	0.44 (-0.19, 1.07)	1.41 (1.32 1.53)
**Elixhauser Tertile**					
Tertile 1 (-4 to 6)					
Day 0-30	173 (0.05)	103 (0.16)	70 (0.02)	0.14 (0.10, 0.17)	7.38 (5.42, 10.05)
Day 0-90	380 (0.10)	167 (0.26)	213 (0.07)	0.19 (0.15, 0.23)	3.94 (3.20, 4.84)
Day 0-180	680 (0.18)	233 (0.36)	447 (0.14)	0.21 (0.16, 0.26)	2.59 (2.20, 3.04)
Day 0-365	1,341 (0.36)	371 (0.57)	970 (0.31)	0.26 (0.19, 0.32)	1.92 (1.70, 2.16)
Tertile 2 (6 to 24)					
Day 0-30	693 (0.18)	343 (0.54)	350 (0.11)	0.43 (0.37, 0.48)	5.27 (4.54, 6.12)
Day 0-90	1,549 (0.41)	555 (0.87)	994 (0.32)	0.55 (0.48, 0.63)	2.99 (2.69, 3.32)
Day 0-180	2,912 (0.77)	810 (1.27)	2,102 (0.67)	0.60 (0.51, 0.69)	2.07 (1.90, 2.24)
Day 0-365	5,610 (1.49)	1,298 (2.03)	4,312 (1.38)	0.66 (0.54, 0.78)	1.61 (1.51, 1.71)
Tertile 3 (24 to 187)					
Day 0-30	3,767 (1.00)	1,240 (2.07)	2,527 (0.80)	1.28 (1.16, 1.40)	3.14 (2.93, 3.37)
Day 0-90	9,256 (2.45)	2,363 (3.95)	6,893 (2.17)	1.78 (1.61, 1.94)	2.11 (2.01, 2.22)
Day 0-180	16,412 (4.35)	3,626 (6.06)	12,786 (4.03)	2.03 (1.83, 2.24)	1.73 (1.67, 1.80)
Day 0-365	28,195 (7.47)	5,534 (9.25)	22,661 (7.14)	2.11 (1.86, 2.36)	1.50 (1.45, 1.54)
**Residence in a Primary Care Shortage Area**					
Yes					

© 2024 Govier DJ et al. *JAMA Network Open*.

	Overall Incidence, n (%)	Incidence in SARS-CoV-2, n (%)	Incidence in Comparators, n (%)	Risk Difference, % (95% CI)	Adjusted Hazard Ratio (95% CI)
Day 0-30	1,168 (0.43)	452 (0.96)	716 (0.32)	0.64 (0.55, 0.73)	3.37 (2.99, 3.79)
Day 0-90	2,702 (0.99)	813 (1.72)	1,889 (0.84)	0.89 (0.76, 1.01)	2.26 (2.08, 2.45)
Day 0-180	4,734 (1.73)	1,200 (2.55)	3,534 (1.57)	0.98 (0.83, 1.13)	1.78 (1.67, 1.90)
Day 0-365	8,397 (3.08)	1,837 (3.90)	6,560 (2.91)	0.99 (0.80, 1.18)	1.47 (1.40, 1.55)
No					
Day 0-30	3,465 (0.40)	1,234 (0.87)	2,231 (0.31)	0.56 (0.51, 0.61)	3.08 (2.87, 3.30)
Day 0-90	8,483 (0.99)	2,272 (1.60)	6,211 (0.87)	0.73 (0.67, 0.80)	2.02 (1.92, 2.12)
Day 0-180	15,270 (1.78)	3,469 (2.44)	11,801 (1.65)	0.80 (0.71, 0.88)	1.61 (1.55, 1.68)
Day 0-365	26,749 (3.11)	5,366 (3.78)	21,383 (2.98)	0.80 (0.69, 0.91)	1.39 (1.35, 1.43)
**COVID-19 Pandemic Wave**					
Wave 1 (Mar-Jun 2020)					
Day 0-30	615 (0.55)	238 (1.28)	377 (0.41)	0.88 (0.71, 1.04)	3.74 (3.17, 4.40)
Day 0-90	1,428 (1.28)	408 (2.20)	1,020 (1.10)	1.10 (0.87, 1.32)	2.28 (2.03, 2.56)
Day 0-180	2,545 (2.27)	596 (3.21)	1,949 (2.10)	1.11 (0.84, 1.38)	1.73 (1.58, 1.90)
Day 0-365	4,396 (3.95)	900 (4.85)	3,496 (3.77)	1.08 (0.74, 1.41)	1.47 (1.37, 1.59)
Wave 2 (Jul-Nov 2020)					
Day 0-30	1,505 (0.38)	502 (0.76)	1,003 (0.30)	0.45 (0.38, 0.52)	2.74 (2.46, 3.05)
Day 0-90	3,667 (0.92)	931 (1.40)	2,736 (0.83)	0.58 (0.48, 0.67)	1.86 (1.72, 2.00)
Day 0-180	6,671 (1.68)	1,471 (2.21)	5,200 (1.57)	0.64 (0.52, 0.77)	1.55 (1.46, 1.64)
Day 0-365	11,866 (2.98)	2,374 (3.57)	9,492 (2.86)	0.71 (0.56, 0.86)	1.37 (1.31, 1.43)
Wave 3 (Dec 2020-Apr 2021)					
Day 0-30	2,513 (0.40)	946 (0.91)	1,567 (0.30)	0.61 (0.55, 0.67)	3.30 (3.04, 3.58)
Day 0-90	6,090 (0.98)	1,746 (1.68)	4,344 (0.84)	0.84 (0.76, 0.92)	2.17 (2.05, 2.29)
Day 0-180	10,788 (1.73)	2,602 (2.50)	8,186 (1.58)	0.92 (0.82, 1.02)	1.71 (1.63, 1.79)
Day 0-365	18,884 (3.03)	3,929 (3.78)	14,955 (2.88)	0.89 (0.77, 1.02)	1.42 (1.37, 1.47)
**Hospitalized at Index**					
Yes					
Day 0-30	1,347 (0.60)	593 (1.59)	754 (0.40)	1.18 (1.05, 1.31)	4.95 (4.35, 5.62)
Day 0-90	3,522 (1.57)	1,384 (3.70)	2,138 (1.15)	2.56 (2.36, 2.76)	3.36 (3.11, 3.62)
Day 0-180	6,148 (2.75)	2,162 (5.78)	3,986 (2.14)	3.65 (3.40, 3.90)	2.70 (2.55, 2.86)
Day 0-365	10,501 (4.69)	3,281 (8.78)	7,220 (3.87)	4.91 (4.61, 5.21)	2.29 (2.19, 2.40)
No					
Day 0-30	2,408 (0.27)	305 (0.20)	2,103 (0.28)	-0.08 (-0.10, -0.05)	1.08 (0.94, 1.23)
Day 0-90	6,785 (0.75)	913 (0.60)	5,872 (0.78)	-0.18 (-0.22, -0.13)	1.11 (1.03, 1.20)
Day 0-180	12,978 (1.43)	1,719 (1.13)	11,259 (1.49)	-0.36 (-0.42, -0.30)	1.06 (1.00, 1.12)
Day 0-365	23,767 (2.62)	3,134 (2.07)	20,633 (2.73)	-0.66 (-0.74, -0.58)	1.05 (1.01, 1.09)

eTable 6 Footnotes: Abbreviations: CI: confidence interval

© 2024 Govier DJ et al. *JAMA Network Open*.

eTable 7. Sample Characteristics for Veterans With SARS-CoV-2 and Matched Comparators, by Age Group

	<65 Years			65-84 Years			85+ Years		
	**Comparator, N = 506,722 (82%)**	**SARS-CoV-2, N = 108,233 (18%)**	**SMD**	**Comparator, N = 390,492 (84%)**	**SARS-CoV-2, N = 72,192 (16%)**	**SMD**	**Comparator, N = 45,870 (84%)**	**SARS-CoV-2, N = 8,711 (16%)**	**SMD**
**Variables Used in Matching**									
Sex, n (%)			0.013			0.03			0.019
Male	418,075 (82.51)	89,812 (82.98)		376,776 (96.49)	70,035 (97.01)		45,128 (98.38)	8,548 (98.13)	
Female	88,647 (17.49)	18,421 (17.02)		13,716 (3.51)	2,157 (2.99)		742 (1.62)	163 (1.87)	
Race, n (%)			0.028			0.015			0.057
American Indian/Alaskan Native	5,413 (1.07)	1,119 (1.03)		3,177 (0.81)	618 (0.86)		251 (0.55)	48 (0.55)	
Asian	7,376 (1.46)	1,578 (1.46)		1,996 (0.51)	312 (0.43)		250 (0.55)	38 (0.44)	
Black/African American	142,832 (28.19)	30,259 (27.96)		72,097 (18.46)	13,313 (18.44)		5,634 (12.28)	1,222 (14.03)	
More than one race	6,498 (1.28)	1,322 (1.22)		3,317 (0.85)	650 (0.90)		261 (0.57)	58 (0.67)	
Native Hawaiian/Pacific Islander	5,442 (1.07)	1,165 (1.08)		3,075 (0.79)	551 (0.76)		339 (0.74)	55 (0.63)	
White	311,350 (61.44)	66,149 (61.12)		305,236 (78.17)	56,430 (78.17)		38,971 (84.96)	7,255 (83.29)	
Missing	27,811 (5.49)	6,641 (6.14)		1,594 (0.41)	318 (0.44)		164 (0.36)	35 (0.40)	
Ethnicity, n (%)			0.014			0.016			0.006
Hispanic/Latino	64,596 (12.75)	14,171 (13.09)		24,107 (6.17)	4,310 (5.97)		2,259 (4.92)	438 (5.03)	
Not Hispanic/Latino	424,388 (83.75)	90,109 (83.25)		354,042 (90.67)	65,761 (91.09)		41,892 (91.33)	7,952 (91.29)	
Missing	17,738 (3.50)	3,953 (3.65)		12,343 (3.16)	2,121 (2.94)		1,719 (3.75)	321 (3.68)	
Rurality, n (%)			0.018			0.003			0.02
Urban	374,490 (73.90)	80,817 (74.67)		253,525 (64.92)	46,781 (64.80)		31,416 (68.49)	5,886 (67.57)	
Not Urban/Missing	132,232 (26.10)	27,416 (25.33)		136,967 (35.08)	25,411 (35.20)		14,454 (31.51)	2,825 (32.43)	
BMI, mean (SD)	32.00 (6.77)	32.24 (6.42)	-0.036	30.84 (6.36)	30.66 (6.06)	0.029	27.14 (4.74)	26.85 (4.71)	0.061
Smoking Status, n (%)			0.030			0.055			0.161
Never Smoker	224,804 (44.36)	49,448 (45.69)		130,535 (33.43)	22,716 (31.47)		17,219 (37.54)	2,950 (33.87)	
Current Smoker	77,572 (15.31)	16,422 (15.17)		40,354 (10.33)	7,360 (10.20)		1,050 (2.29)	289 (3.32)	
Former Smoker	168,740 (33.30)	35,268 (32.59)		205,478 (52.62)	38,943 (53.94)		25,278 (55.11)	4,726 (54.25)	
Missing	35,606 (7.03)	7,095 (6.56)		14,125 (3.62)	3,173 (4.40)		2,323 (5.06)	746 (8.56)	

© 2024 Govier DJ et al. *JAMA Network Open*.

	<65 Years			65-84 Years			85+ Years		
	**Comparator, N = 506,722 (82%)**	**SARS-CoV-2, N = 108,233 (18%)**	**SMD**	**Comparator, N = 390,492 (84%)**	**SARS-CoV-2, N = 72,192 (16%)**	**SMD**	**Comparator, N = 45,870 (84%)**	**SARS-CoV-2, N = 8,711 (16%)**	**SMD**
Nosos Risk-Adjustment Score Category, n (%)*			0.062			0.074			0.206
Category 1 (0, 0.417)	17,282 (3.41)	4,234 (3.91)		3,816 (0.98)	633 (0.88)		2,448 (5.34)	391 (4.49)	
Category 2 (0.417, 0.471)	26,380 (5.21)	6,385 (5.90)		12,184 (3.12)	2,001 (2.77)		2,707 (5.90)	437 (5.02)	
Category 3 (0.471, 0.534)	35,475 (7.00)	8,064 (7.45)		16,998 (4.35)	2,939 (4.07)		2,967 (6.47)	471 (5.41)	
Category 4 (0.534, 0.611)	43,613 (8.61)	9,539 (8.81)		21,786 (5.58)	3,890 (5.39)		3,598 (7.84)	504 (5.79)	
Category 5 (0.611, 0.707)	50,317 (9.93)	10,760 (9.94)		27,699 (7.09)	4,884 (6.77)		3,948 (8.61)	622 (7.14)	
Category 6 (0.707, 0.829)	56,627 (11.18)	12,043 (11.13)		35,260 (9.03)	6,109 (8.46)		4,319 (9.42)	676 (7.76)	
Category 7 (0.829, 0.998)	60,821 (12.00)	12,774 (11.80)		43,942 (11.25)	7,782 (10.78)		4,569 (9.96)	827 (9.49)	
Category 8 (0.998, 1.259)	63,212 (12.47)	13,561 (12.53)		56,355 (14.43)	9,862 (13.66)		4,953 (10.80)	961 (11.03)	
Category 9 (1.259, 1.805)	65,155 (12.86)	13,719 (12.68)		71,885 (18.41)	13,315 (18.44)		5,937 (12.94)	1,245 (14.29)	
Category 10 (1.805, 39.370)	70,210 (13.86)	14,114 (13.04)		96,349 (24.67)	19,784 (27.40)		9,881 (21.54)	2,345 (26.92)	
Missing	17,630 (3.48)	3,040 (2.81)		4,218 (1.08)	993 (1.38)		543 (1.18)	232 (2.66)	
CAN score Category, n (%)			0.049			0.076			0.241
Category 1 (0, 20)	127,182 (25.10)	27,433 (25.35)		27,671 (7.09)	5,024 (6.96)		9 (0.02)	2 (0.02)	
Category 2 (25, 40)	95,194 (18.79)	21,164 (19.55)		50,006 (12.81)	8,328 (11.54)		396 (0.86)	49 (0.56)	
Category 3 (45, 60)	99,942 (19.72)	21,836 (20.17)		72,513 (18.57)	12,368 (17.13)		4,654 (10.15)	553 (6.35)	
Category 4 (65, 80)	98,944 (19.53)	21,051 (19.45)		102,992 (26.37)	19,010 (26.33)		12,752 (27.80)	1,960 (22.50)	
Category 5 (85, 90)	47,657 (9.40)	9,591 (8.86)		79,317 (20.31)	15,228 (21.09)		12,140 (26.47)	2,415 (27.72)	
Category 6 (95, 99)	23,688 (4.67)	4,808 (4.44)		53,829 (13.78)	11,222 (15.54)		15,456 (33.70)	3,496 (40.13)	
Missing	14,115 (2.79)	2,350 (2.17)		4,164 (1.07)	1,012 (1.40)		463 (1.01)	236 (2.71)	
Gagne Index Score, mean (SD)	0.78 (1.62)	0.76 (1.63)	0.010	2.06 (2.57)	2.16 (2.74)	-0.039	2.66 (2.78)	2.97 (2.95)	-0.110
# VHA Primary Care Visits in Previous 24 Months, mean (SD)	6.80 (8.49)	7.08 (7.85)	-0.035	10.04 (11.94)	10.33 (11.13)	-0.026	9.64 (12.70)	10.52 (12.14)	-0.071
# VHA Inpatient Admissions in Previous 24 Months, mean (SD)	0.23 (1.00)	0.22 (0.96)	0.014	0.48 (1.48)	0.51 (1.42)	-0.020	0.48 (1.51)	0.62 (1.52)	-0.092

© 2024 Govier DJ et al. *JAMA Network Open*.

	<65 Years			65-84 Years			85+ Years		
	**Comparator, N = 506,722 (82%)**	**SARS-CoV-2, N = 108,233 (18%)**	**SMD**	**Comparator, N = 390,492 (84%)**	**SARS-CoV-2, N = 72,192 (16%)**	**SMD**	**Comparator, N = 45,870 (84%)**	**SARS-CoV-2, N = 8,711 (16%)**	**SMD**
# VHA Specialty Care Visits in Previous 24 Months, mean (SD)	10.86 (12.89)	11.22 (11.81)	-0.029	16.78 (17.47)	17.59 (16.20)	-0.049	13.50 (15.29)	14.86 (14.89)	-0.090
COVID-19 Vaccination Before Index Date, n (%)			0.007			0.010			0.049
Vaccinated	5,231 (1.03)	1,046 (0.97)		7,127 (1.83)	1,403 (1.94)		1,172 (2.56)	294 (3.38)	
Not Vaccinated	170,116 (33.57)	36,460 (33.69)		125,488 (32.14)	23,031 (31.90)		13,451 (29.32)	2,494 (28.63)	
Vaccine Not Available	331,375 (65.40)	70,727 (65.35)		257,877 (66.04)	47,758 (66.15)		31,247 (68.12)	5,923 (67.99)	
CDC COVID-19 High-Risk Conditions, n (%)									
Cancer	20,824 (4.11)	4,109 (3.80)	0.016	74,299 (19.03)	12,642 (17.51)	0.039	10,786 (23.51)	1,840 (21.12)	0.057
Pulmonary	81,276 (16.04)	17,200 (15.89)	0.004	131,037 (33.56)	24,288 (33.64)	-0.002	15,857 (34.57)	3,055 (35.07)	-0.011
Hypertension	230,188 (45.43)	50,532 (46.69)	-0.025	334,614 (85.69)	61,282 (84.89)	0.023	41,195 (89.81)	7,736 (88.81)	0.032
Diabetes	114,814 (22.66)	25,853 (23.89)	-0.029	200,166 (51.26)	36,531 (50.60)	0.013	19,861 (43.30)	3,639 (41.77)	0.031
Dementia	5,722 (1.13)	925 (0.85)	0.028	28,812 (7.38)	5,633 (7.80)	-0.016	10,944 (23.86)	2,312 (26.54)	-0.062
Coronary Heart Disease	65,800 (12.99)	14,165 (13.09)	-0.003	174,386 (44.66)	33,236 (46.04)	-0.028	25,965 (56.61)	5,047 (57.94)	-0.027
Sickle Cell	1,199 (0.24)	253 (0.23)	0.001	456 (0.12)	105 (0.15)	-0.008	18 (0.04)	6 (0.07)	-0.013
Transplant	1,439 (0.28)	300 (0.28)	0.001	1,631 (0.42)	294 (0.41)	0.002	35 (0.08)	5 (0.06)	0.007
Stroke/Cerebrovascular Disease	14,765 (2.91)	2,825 (2.61)	0.019	36,390 (9.32)	7,151 (9.91)	-0.020	5,720 (12.47)	1,260 (14.46)	-0.058
Liver Disease	47,094 (9.29)	10,596 (9.79)	-0.017	48,924 (12.53)	8,697 (12.05)	0.015	2,578 (5.62)	433 (4.97)	0.029
Kidney Disease	64,722 (12.77)	14,216 (13.13)	-0.011	126,632 (32.43)	23,866 (33.06)	-0.013	18,878 (41.16)	3,869 (44.42)	-0.066
Congestive Heart Failure	22,397 (4.42)	4,551 (4.20)	0.011	62,977 (16.13)	12,431 (17.22)	-0.029	10,623 (23.16)	2,257 (25.91)	-0.064
Major Depression Diagnosis	200,394 (39.55)	41,013 (37.89)	0.034	97,639 (25.00)	18,908 (26.19)	-0.027	6,629 (14.45)	1,489 (17.09)	-0.073
Anxiety Diagnosis	151,344 (29.87)	31,172 (28.80)	0.023	58,023 (14.86)	11,185 (15.49)	-0.018	4,119 (8.98)	879 (10.09)	-0.038
PTSD Diagnosis	162,257 (32.02)	33,144 (30.62)	0.030	76,391 (19.56)	14,807 (20.51)	-0.024	2,139 (4.66)	563 (6.46)	-0.079
Substance Use Disorder	82,794 (16.34)	17,091 (15.79)	0.015	34,052 (8.72)	6,563 (9.09)	-0.013	824 (1.80)	191 (2.19)	-0.028
Bipolar Diagnosis	27,557 (5.44)	5,410 (5.00)	0.020	8,499 (2.18)	1,849 (2.56)	-0.025	245 (0.53)	63 (0.72)	-0.024
Schizophrenia Diagnosis	13,035 (2.57)	2,172 (2.01)	0.038	7,518 (1.93)	1,922 (2.66)	-0.049	251 (0.55)	87 (1.00)	-0.052

© 2024 Govier DJ et al. *JAMA Network Open*.

	<65 Years			65-84 Years			85+ Years		
	**Comparator, N = 506,722 (82%)**	**SARS-CoV-2, N = 108,233 (18%)**	**SMD**	**Comparator, N = 390,492 (84%)**	**SARS-CoV-2, N = 72,192 (16%)**	**SMD**	**Comparator, N = 45,870 (84%)**	**SARS-CoV-2, N = 8,711 (16%)**	**SMD**
Immunocompromised	42,926 (8.47)	8,886 (8.21)	0.009	45,378 (11.62)	8,785 (12.17)	-0.017	3,594 (7.84)	766 (8.79)	-0.035
Community Living Center at Index, n (%)	2,301 (0.45)	429 (0.40)	0.009	5,279 (1.35)	1,187 (1.64)	-0.024	953 (2.08)	277 (3.18)	-0.069
Distance to Nearest VAMC, mean (SD), miles	33.99 (34.21)	33.56 (35.61)	0.013	37.91 (35.88)	37.43 (37.10)	0.013	36.67 (36.51)	35.38 (37.41)	0.035
**Other Variables (not used in matching)**									
Non-Preventable Hospitalization Count, 1-year Post-index, mean (SD)	0.15 (0.57)	0.30 (0.88)	-0.208	0.37 (0.90)	0.75 (1.24)	-0.351	0.51 (0.97)	0.95 (1.21)	-0.405
COVID-19 Pandemic Wave of Index, n (%)			0.008			0.013			0.021
First (March-June 2020)	48,965 (9.66)	10,708 (9.89)		37,801 (9.68)	6,722 (9.31)		6,000 (13.08)	1,146 (13.16)	
Second (July-Nov 2020)	179,962 (35.51)	38,336 (35.42)		136,088 (34.85)	25,290 (35.03)		15,457 (33.70)	2,852 (32.74)	
Third (Dec 2020-April 2021)	277,795 (54.82)	59,189 (54.69)		216,603 (55.47)	40,180 (55.66)		24,413 (53.22)	4,713 (54.10)	
Residence in Primary Care Shortage Area, n (%)	120,059 (23.69)	26,816 (24.78)	0.025	95,107 (24.36)	18,193 (25.20)	0.020	10,685 (23.29)	2,138 (24.54)	0.029
Medicare Advantage at Index, n (%)	41,197 (8.13)	8,110 (7.49)	0.024	114,616 (29.35)	23,373 (32.38)	0.066	15,969 (34.81)	3,707 (42.56)	0.159
Long-term Institutionalization During Baseline or Follow-up, n (%)	3,355 (0.66)	997 (0.92)	-0.029	26,035 (6.67)	7,679 (10.64)	-0.142	10,737 (23.41)	2,831 (32.50)	-0.204
Elixhauser Score, mean (SD)	13.46 (17.35)	13.07 (17.39)	0.022	29.02 (24.79)	29.13 (25.70)	-0.004	35.06 (25.67)	35.27 (25.62)	-0.008
Elixhauser score tertiles, n (%)*			0.025			0.024			0.032
Tertile 1 (-4, 6)	234,442 (46.27)	50,597 (46.75)		71,535 (18.32)	13,790 (19.10)		5,986 (13.05)	1,057 (12.13)	
Tertile 2 (6, 24)	174,181 (34.37)	37,756 (34.88)		127,267 (32.59)	23,720 (32.86)		12,102 (26.38)	2,381 (27.33)	
Tertile 3 (24, 187)	98,099 (19.36)	19,880 (18.37)		191,690 (49.09)	34,682 (48.04)		27,782 (60.57)	5,273 (60.53)	

eTable 7 Footnotes: Pregnancy was a matching variable but was zero for all persons. State of residence was a matching variable and included 50 states and Washington D.C.; Index Month was a matching variable and spanned 14 months (not shown, both SMDs <0.1). Race and ethnicity data from the VA electronic health record are collected through self-identification either at enrollment or at a health care encounter. Abbreviations: SMD: standardized mean difference; CAN: Care Assessment of Need; CDC: Centers for Disease Control and Prevention; VHA: Veterans Health Administration; SD: standard deviation

*Overlapping score categories are mutually exclusive.

© 2024 Govier DJ et al. *JAMA Network Open*.

eTable 8. Sample Characteristics for Veterans With SARS-CoV-2 and Matched Comparators, by Sex

	Male Sex			Female Sex		
	Comparator, N = 839,979 (83%)	SARS-CoV-2, N = 168,395 (17%)	SMD	Comparator, N = 103,105 (83%)	SARS-CoV-2, N = 20,741 (17%)	SMD
**Variables Used in Matching**						
Age Group, n (%)			0.071			0.090
<65	418,075 (49.77)	89,812 (53.33)		88,647 (85.98)	18,421 (88.81)	
65-84	376,776 (44.86)	70,035 (41.59)		13,716 (13.30)	2,157 (10.40)	
85+	45,128 (5.37)	8,548 (5.08)		742 (0.72)	163 (0.79)	
Race, n (%)			0.035			0.042
American Indian/Alaskan Native	7,654 (0.91)	1,518 (0.90)		1,187 (1.15)	267 (1.29)	
Asian	8,309 (0.99)	1,656 (0.98)		1,313 (1.27)	272 (1.31)	
Black/African American	183,352 (21.83)	37,585 (22.32)		37,211 (36.09)	7,209 (34.76)	
More than one race	8,481 (1.01)	1,744 (1.04)		1,595 (1.55)	286 (1.38)	
Native Hawaiian/Pacific Islander	7,716 (0.92)	1,551 (0.92)		1,140 (1.11)	220 (1.06)	
White	599,816 (71.41)	118,467 (70.35)		55,741 (54.06)	11,367 (54.80)	
Missing	24,651 (2.93)	5,874 (3.49)		4,918 (4.77)	1,120 (5.40)	
Ethnicity, n (%)			0.012			0.015
Hispanic/Latino	80,003 (9.52)	16,650 (9.89)		10,959 (10.63)	2,269 (10.94)	
Not Hispanic/Latino	732,026 (87.15)	146,167 (86.80)		88,296 (85.64)	17,655 (85.12)	
Missing	27,950 (3.33)	5,578 (3.31)		3,850 (3.73)	817 (3.94)	
Rurality, n (%)			0.017			0.011
Urban	580,433 (69.10)	117,689 (69.89)		78,998 (76.62)	15,795 (76.15)	
Not Urban/Missing	259,546 (30.90)	50,706 (30.11)		24,107 (23.38)	4,946 (23.85)	
BMI, mean (SD)	31.26 (6.55)	31.35 (6.26)	-0.015	31.53 (7.04)	31.72 (6.91)	-0.027
Smoking Status, n (%)			0.018			0.019
Never Smoker	313,856 (37.36)	63,139 (37.49)		58,702 (56.93)	11,975 (57.74)	
Current Smoker	108,753 (12.95)	22,103 (13.13)		10,223 (9.92)	1,968 (9.49)	
Former Smoker	371,259 (44.20)	73,326 (43.54)		28,237 (27.39)	5,611 (27.05)	
Missing	46,111 (5.49)	9,827 (5.84)		5,943 (5.76)	1,187 (5.72)	
Nosos Risk-Adjustment Score Category, n (%)*			0.035			0.034

© 2024 Govier DJ et al. *JAMA Network Open*.

	Male Sex			Female Sex		
Category 1 (0, 0.417)	21,395 (2.55)	4,780 (2.84)		2,151 (2.09)	478 (2.30)	
Category 2 (0.417, 0.471)	37,523 (4.47)	8,077 (4.80)		3,748 (3.64)	746 (3.60)	
Category 3 (0.471, 0.534)	49,840 (5.93)	10,280 (6.10)		5,600 (5.43)	1,194 (5.76)	
Category 4 (0.534, 0.611)	61,502 (7.32)	12,462 (7.40)		7,495 (7.27)	1,471 (7.09)	
Category 5 (0.611, 0.707)	72,932 (8.68)	14,411 (8.56)		9,032 (8.76)	1,855 (8.94)	
Category 6 (0.707, 0.829)	84,959 (10.11)	16,660 (9.89)		11,247 (10.91)	2,168 (10.45)	
Category 7 (0.829, 0.998)	96,378 (11.47)	18,745 (11.13)		12,954 (12.56)	2,638 (12.72)	
Category 8 (0.998, 1.259)	109,808 (13.07)	21,376 (12.69)		14,712 (14.27)	3,008 (14.50)	
Category 9 (1.259, 1.805)	126,309 (15.04)	24,855 (14.76)		16,668 (16.17)	3,424 (16.51)	
Category 10 (1.805, 39.370)	159,351 (18.97)	32,957 (19.57)		17,089 (16.57)	3,286 (15.84)	
Missing	19,982 (2.38)	3,792 (2.25)		2,409 (2.34)	473 (2.28)	
CAN score Category, n (%)			0.036			0.028
Category 1 (0, 20)	137,638 (16.39)	29,140 (17.30)		17,224 (16.71)	3,319 (16.00)	
Category 2 (25, 40)	127,187 (15.14)	25,835 (15.34)		18,409 (17.85)	3,706 (17.87)	
Category 3 (45, 60)	153,800 (18.31)	30,000 (17.82)		23,309 (22.61)	4,757 (22.94)	
Category 4 (65, 80)	188,331 (22.42)	36,557 (21.71)		26,357 (25.56)	5,464 (26.34)	
Category 5 (85, 90)	127,313 (15.16)	24,923 (14.80)		11,801 (11.45)	2,311 (11.14)	
Category 6 (95, 99)	88,596 (10.55)	18,683 (11.09)		4,377 (4.25)	843 (4.06)	
Missing	17,114 (2.04)	3,257 (1.93)		1,628 (1.58)	341 (1.64)	
Gagne Index Score, mean (SD)	1.48 (2.29)	1.48 (2.38)	<0.001	0.75 (1.49)	0.75 (1.51)	0.003
# VHA Primary Care Visits in Previous 24 Months, mean (SD)	8.23 (10.46)	8.42 (9.65)	-0.018	8.65 (9.88)	8.99 (9.07)	-0.036
# VHA Inpatient Admissions in Previous 24 Months, mean (SD)	0.36 (1.29)	0.37 (1.24)	-0.003	0.20 (0.89)	0.18 (0.80)	0.024
# VHA Specialty Care Visits in Previous 24 Months, mean (SD)	13.48 (15.52)	13.85 (14.27)	-0.025	13.13 (13.81)	13.59 (12.93)	-0.034
COVID-19 Vaccination Before Index Date, n (%)			0.001			0.002
Vaccinated	12,364 (1.47)	2,504 (1.49)		1,166 (1.13)	239 (1.15)	
Not Vaccinated	274,256 (32.65)	54,977 (32.65)		34,799 (33.75)	7,008 (33.79)	
Vaccine Not Available	553,359 (65.88)	110,914 (65.87)		67,140 (65.12)	13,494 (65.06)	
CDC COVID-19 High-Risk Conditions, n (%)						
Cancer	100,929 (12.02)	17,733 (10.53)	0.047	4,980 (4.83)	858 (4.14)	0.034

© 2024 Govier DJ et al. *JAMA Network Open*.

	Male Sex			Female Sex		
Pulmonary	206,093 (24.54)	40,153 (23.84)	0.016	22,077 (21.41)	4,390 (21.17)	0.006
Hypertension	565,412 (67.31)	111,602 (66.27)	0.022	40,585 (39.36)	7,948 (38.32)	0.021
Diabetes	315,306 (37.54)	62,168 (36.92)	0.013	19,535 (18.95)	3,855 (18.59)	0.009
Dementia	43,889 (5.23)	8,564 (5.09)	0.006	1,589 (1.54)	306 (1.48)	0.005
Coronary Heart Disease	255,224 (30.38)	50,267 (29.85)	0.012	10,927 (10.60)	2,181 (10.52)	0.003
Sickle Cell	1,299 (0.15)	288 (0.17)	-0.004	374 (0.36)	76 (0.37)	-0.001
Transplant	2,972 (0.35)	574 (0.34)	0.002	133 (0.13)	25 (0.12)	0.002
Stroke/Cerebrovascular Disease	53,805 (6.41)	10,645 (6.32)	0.003	3,070 (2.98)	591 (2.85)	0.008
Liver Disease	91,740 (10.92)	18,321 (10.88)	0.001	6,856 (6.65)	1,405 (6.77)	-0.005
Kidney Disease	200,012 (23.81)	39,935 (23.72)	0.002	10,220 (9.91)	2,016 (9.72)	0.006
Congestive Heart Failure	92,542 (11.02)	18,607 (11.05)	-0.001	3,455 (3.35)	632 (3.05)	0.017
Major Depression Diagnosis	253,141 (30.14)	50,985 (30.28)	-0.003	51,521 (49.97)	10,425 (50.26)	-0.006
Anxiety Diagnosis	173,551 (20.66)	35,128 (20.86)	-0.005	39,935 (38.73)	8,108 (39.09)	-0.007
PTSD Diagnosis	204,907 (24.39)	41,381 (24.57)	-0.004	35,880 (34.80)	7,133 (34.39)	0.009
Substance Use Disorder	108,327 (12.90)	21,965 (13.04)	-0.004	9,343 (9.06)	1,880 (9.06)	<0.001
Bipolar Diagnosis	28,537 (3.40)	5,819 (3.46)	-0.003	7,764 (7.53)	1,503 (7.25)	0.011
Schizophrenia Diagnosis	18,604 (2.21)	3,778 (2.24)	-0.002	2,200 (2.13)	403 (1.94)	0.013
Immunocompromised	81,657 (9.72)	16,382 (9.73)	<0.001	10,241 (9.93)	2,055 (9.91)	0.001
Community Living Center at Index, n (%)	8,127 (0.97)	1,811 (1.08)	-0.011	406 (0.39)	82 (0.40)	<0.001
Distance to Nearest VAMC, mean (SD), miles	36.08 (35.25)	35.29 (36.46)	0.022	32.99 (33.53)	33.70 (35.11)	-0.021
**Other Variables (not used in matching)**						
Non-Preventable Hospitalization Count, 1-year Post-index, mean (SD)	0.27 (0.78)	0.53 (1.10)	-0.274	0.14 (0.52)	0.26 (0.75)	-0.183
COVID-19 Pandemic Wave of Index, n (%)			<0.001			0.003
First (March-June 2020)	82,704 (9.85)	16,559 (9.83)		10,062 (9.76)	2,017 (9.72)	
Second (July-Nov 2020)	295,422 (35.17)	59,242 (35.18)		36,085 (35.00)	7,236 (34.89)	
Third (Dec 2020-April 2021)	461,853 (54.98)	92,594 (54.99)		56,958 (55.24)	11,488 (55.39)	
Residence in Primary Care Shortage Area, n (%)	202,859 (24.15)	42,323 (25.13)	0.023	22,992 (22.30)	4,824 (23.26)	0.023
Medicare Advantage at Index, n (%)	162,476 (19.34)	33,445 (19.86)	0.013	9,306 (9.03)	1,745 (8.41)	0.022
Long-term Institutionalization During Baseline or Follow-up, n (%)	38,937 (4.64)	11,172 (6.63)	-0.087	1,190 (1.15)	335 (1.62)	-0.039

© 2024 Govier DJ et al. *JAMA Network Open*.

	Male Sex			Female Sex		
Elixhauser Score, mean (SD)	21.93 (23.18)	21.19 (23.45)	0.032	12.93 (16.20)	12.36 (15.99)	0.036
Elixhauser score tertiles, n (%)*			0.046			0.040
Tertile 1 (-4, 6)	265,068 (31.56)	55,784 (33.13)		46,895 (45.48)	9,660 (46.57)	
Tertile 2 (6, 24)	275,503 (32.80)	56,119 (33.33)		38,047 (36.90)	7,738 (37.31)	
Tertile 3 (24, 187)	299,408 (35.64)	56,492 (33.55)		18,163 (17.62)	3,343 (16.12)	

eTable 8 Footnotes: Pregnancy was a matching variable but was zero for all persons. State of residence was a matching variable and included 50 states and Washington D.C.; Index Month was a matching variable and spanned 14 months (not shown, both SMDs <0.1). Race and ethnicity data from the VA electronic health record are collected through self-identification either at enrollment or at a health care encounter.: SMD: standardized mean difference; CAN: Care Assessment of Need; CDC: Centers for Disease Control and Prevention; VHA: Veterans Health Administration; SD: standard deviation

*Overlapping score categories are mutually exclusive.

© 2024 Govier DJ et al. *JAMA Network Open*.

eTable 9. Sample Characteristics for Veterans With SARS-CoV-2 and Matched Comparators, by Elixhauser Rehospitalization Score Tertile

	Tertile 1 (-4, 6)			Tertile 2 (6, 24)			Tertile 3 (24, 187)		
	Comparator, N = 311,963 (83%)	SARS-CoV-2, N = 65,444 (17%)	SMD	Comparator, N = 313,550 (83%)	SARS-CoV-2, N = 63,857 (17%)	SMD	Comparator, N = 317,571 (84%)	SARS-CoV-2, N = 59,835 (16%)	SMD
**Variables Used in Matching**									
Age Group, n (%)			0.052			0.073			0.052
<65	234,442 (75.15)	50,597 (77.31)		174,181 (55.55)	37,756 (59.13)		98,099 (30.89)	19,880 (33.22)	
65-84	71,535 (22.93)	13,790 (21.07)		127,267 (40.59)	23,720 (37.15)		191,690 (60.36)	34,682 (57.96)	
85+	5,986 (1.92)	1,057 (1.62)		12,102 (3.86)	2,381 (3.73)		27,782 (8.75)	5,273 (8.81)	
Sex, n (%)			0.008			0.001			0.006
Male	265,068 (84.97)	55,784 (85.24)		275,503 (87.87)	56,119 (87.88)		299,408 (94.28)	56,492 (94.41)	
Female	46,895 (15.03)	9,660 (14.76)		38,047 (12.13)	7,738 (12.12)		18,163 (5.72)	3,343 (5.59)	
Race, n (%)			0.030			0.033			0.033
American Indian/Alaskan Native	2,929 (0.94)	579 (0.88)		3,116 (0.99)	660 (1.03)		2,796 (0.88)	546 (0.91)	
Asian	4,706 (1.51)	997 (1.52)		3,178 (1.01)	640 (1.00)		1,738 (0.55)	291 (0.49)	
Black/African American	67,815 (21.74)	14,116 (21.57)		73,516 (23.45)	15,206 (23.81)		79,232 (24.95)	15,472 (25.86)	
More than one race	3,665 (1.17)	715 (1.09)		3,355 (1.07)	707 (1.11)		3,056 (0.96)	608 (1.02)	
Native Hawaiian/Pacific Islander	3,241 (1.04)	697 (1.07)		2,997 (0.96)	593 (0.93)		2,618 (0.82)	481 (0.80)	
White	213,216 (68.35)	44,468 (67.95)		217,822 (69.47)	43,757 (68.52)		224,519 (70.70)	41,609 (69.54)	
Missing	16,391 (5.25)	3,872 (5.92)		9,566 (3.05)	2,294 (3.59)		3,612 (1.14)	828 (1.38)	
Ethnicity, n (%)			0.003			0.013			0.016
Hispanic/Latino	38,503 (12.34)	8,110 (12.39)		31,212 (9.95)	6,592 (10.32)		21,247 (6.69)	4,217 (7.05)	
Not Hispanic/Latino	261,404 (83.79)	54,781 (83.71)		272,249 (86.83)	55,167 (86.39)		286,669 (90.27)	53,874 (90.04)	
Missing	12,056 (3.86)	2,553 (3.90)		10,089 (3.22)	2,098 (3.29)		9,655 (3.04)	1,744 (2.91)	
Rurality, n (%)			0.017			0.005			0.018

© 2024 Govier DJ et al. *JAMA Network Open*.

	Tertile 1 (-4, 6)			Tertile 2 (6, 24)			Tertile 3 (24, 187)		
	**Comparator, N = 311,963 (83%)**	**SARS-CoV-2, N = 65,444 (17%)**	**SMD**	**Comparator, N = 313,550 (83%)**	**SARS-CoV-2, N = 63,857 (17%)**	**SMD**	**Comparator, N = 317,571 (84%)**	**SARS-CoV-2, N = 59,835 (16%)**	**SMD**
Urban	223,294 (71.58)	47,357 (72.36)		217,831 (69.47)	44,500 (69.69)		218,306 (68.74)	41,627 (69.57)	
Not Urban/Missing	88,669 (28.42)	18,087 (27.64)		95,719 (30.53)	19,357 (30.31)		99,265 (31.26)	18,208 (30.43)	
BMI, mean (SD)	31.37 (6.22)	31.62 (5.94)	-0.041	31.51 (6.58)	31.67 (6.24)	-0.024	30.99 (6.98)	30.84 (6.81)	0.021
Smoking Status, n (%)			0.059			0.042			0.082
Never Smoker	143,614 (46.04)	30,412 (46.47)		127,143 (40.55)	26,088 (40.85)		101,801 (32.06)	18,614 (31.11)	
Current Smoker	30,903 (9.91)	7,038 (10.75)		41,309 (13.17)	8,027 (12.57)		46,764 (14.73)	9,006 (15.05)	
Former Smoker	106,452 (34.12)	22,526 (34.42)		134,287 (42.83)	27,070 (42.39)		158,757 (49.99)	29,341 (49.04)	
Missing	30,994 (9.94)	5,468 (8.36)		10,811 (3.45)	2,672 (4.18)		10,249 (3.23)	2,874 (4.80)	
Nosos Risk-Adjustment Score Category, n (%)*			0.058			0.040			0.109
Category 1 (0, 0.417)	18,083 (5.80)	3,934 (6.01)		3,612 (1.15)	950 (1.49)		1,851 (0.58)	374 (0.63)	
Category 2 (0.417, 0.471)	28,090 (9.00)	6,272 (9.58)		9,073 (2.89)	1,947 (3.05)		4,108 (1.29)	604 (1.01)	
Category 3 (0.471, 0.534)	34,647 (11.11)	7,500 (11.46)		14,725 (4.70)	3,056 (4.79)		6,068 (1.91)	918 (1.53)	
Category 4 (0.534, 0.611)	39,406 (12.63)	8,256 (12.62)		21,287 (6.79)	4,429 (6.94)		8,304 (2.61)	1,248 (2.09)	
Category 5 (0.611, 0.707)	40,958 (13.13)	8,494 (12.98)		29,139 (9.29)	5,891 (9.23)		11,867 (3.74)	1,881 (3.14)	
Category 6 (0.707, 0.829)	40,311 (12.92)	8,403 (12.84)		38,112 (12.15)	7,530 (11.79)		17,783 (5.60)	2,895 (4.84)	
Category 7 (0.829, 0.998)	36,833 (11.81)	7,710 (11.78)		46,254 (14.75)	9,189 (14.39)		26,245 (8.26)	4,484 (7.49)	
Category 8 (0.998, 1.259)	30,322 (9.72)	6,396 (9.77)		53,532 (17.07)	10,862 (17.01)		40,666 (12.81)	7,126 (11.91)	
Category 9 (1.259, 1.805)	20,302 (6.51)	4,389 (6.71)		56,065 (17.88)	11,508 (18.02)		66,610 (20.97)	12,382 (20.69)	
Category 10 (1.805, 39.370)	7,233 (2.32)	1,511 (2.31)		37,231 (11.87)	7,424 (11.63)		131,976 (41.56)	27,308 (45.64)	
Missing	15,778 (5.06)	2,579 (3.94)		4,520 (1.44)	1,071 (1.68)		2,093 (0.66)	615 (1.03)	
CAN score Category, n (%)			0.042			0.035			0.099
Category 1 (0, 20)	117,185 (37.56)	24,424 (37.32)		33,100 (10.56)	7,272 (11.39)		4,577 (1.44)	763 (1.28)	
Category 2 (25, 40)	77,999 (25.00)	16,459 (25.15)		53,165 (16.96)	10,901 (17.07)		14,432 (4.54)	2,181 (3.65)	
Category 3 (45, 60)	61,629 (19.76)	13,223 (20.21)		80,813 (25.77)	15,873 (24.86)		34,667 (10.92)	5,661 (9.46)	

© 2024 Govier DJ et al. *JAMA Network Open*.

	Tertile 1 (-4, 6)			Tertile 2 (6, 24)			Tertile 3 (24, 187)		
	**Comparator, N = 311,963 (83%)**	**SARS-CoV-2, N = 65,444 (17%)**	**SMD**	**Comparator, N = 313,550 (83%)**	**SARS-CoV-2, N = 63,857 (17%)**	**SMD**	**Comparator, N = 317,571 (84%)**	**SARS-CoV-2, N = 59,835 (16%)**	**SMD**
Category 4 (65, 80)	36,023 (11.55)	7,751 (11.84)		95,529 (30.47)	19,395 (30.37)		83,136 (26.18)	14,875 (24.86)	
Category 5 (85, 90)	6,493 (2.08)	1,415 (2.16)		38,349 (12.23)	7,926 (12.41)		94,272 (29.69)	17,893 (29.90)	
Category 6 (95, 99)	727 (0.23)	155 (0.24)		8,494 (2.71)	1,592 (2.49)		83,752 (26.37)	17,779 (29.71)	
Missing	11,907 (3.82)	2,017 (3.08)		4,100 (1.31)	898 (1.41)		2,735 (0.86)	683 (1.14)	
Gagne Index Score, mean (SD)	-0.14 (0.59)	-0.14 (0.60)	0.003	0.89 (1.01)	0.90 (0.99)	-0.005	3.41 (2.61)	3.62 (2.78)	-0.077
# VHA Primary Care Visits in Previous 24 Months, mean (SD)	4.44 (5.39)	5.02 (5.41)	-0.107	7.50 (8.16)	7.74 (7.50)	-0.030	12.81 (13.83)	13.07 (12.80)	-0.019
# VHA Inpatient Admissions in Previous 24 Months, mean (SD)	0.05 (0.32)	0.04 (0.25)	0.003	0.16 (0.64)	0.14 (0.49)	0.026	0.83 (1.95)	0.90 (1.94)	-0.035
# VHA Specialty Care Visits in Previous 24 Months, mean (SD)	6.97 (8.07)	7.77 (7.66)	-0.101	12.16 (12.71)	12.44 (10.79)	-0.023	21.05 (19.35)	21.92 (18.29)	-0.046
COVID-19 Vaccination Before Index Date, n (%)			0.014			0.012			0.026
Vaccinated	3,736 (1.20)	696 (1.06)		4,393 (1.40)	819 (1.28)		5,401 (1.70)	1,228 (2.05)	
Not Vaccinated	104,188 (33.40)	21,704 (33.16)		102,891 (32.81)	21,145 (33.11)		101,976 (32.11)	19,136 (31.98)	
Vaccine Not Available	204,039 (65.40)	43,044 (65.77)		206,266 (65.78)	41,893 (65.60)		210,194 (66.19)	39,471 (65.97)	
CDC COVID-19 High-Risk Conditions, n (%)									
Cancer	0 (0.00)	0 (0.00)	<0.001	20,526 (6.55)	3,843 (6.02)	0.022	85,383 (26.89)	14,748 (24.65)	0.051
Pulmonary	5,753 (1.84)	1,341 (2.05)	-0.015	78,941 (25.18)	16,407 (25.69)	-0.012	143,476 (45.18)	26,795 (44.78)	0.008
Hypertension	127,871 (40.99)	27,300 (41.72)	-0.015	200,574 (63.97)	40,146 (62.87)	0.023	277,552 (87.40)	52,104 (87.08)	0.010
Diabetes	26,660 (8.55)	5,664 (8.65)	-0.004	109,580 (34.95)	22,802 (35.71)	-0.016	198,601 (62.54)	37,557 (62.77)	-0.005
Dementia	3,124 (1.00)	490 (0.75)	0.027	10,390 (3.31)	1,753 (2.75)	0.033	31,964 (10.07)	6,627 (11.08)	-0.033
Coronary Heart Disease	26,504 (8.50)	5,616 (8.58)	-0.003	68,378 (21.81)	13,707 (21.47)	0.008	171,269 (53.93)	33,125 (55.36)	-0.029
Sickle Cell	370 (0.12)	75 (0.11)	0.001	493 (0.16)	106 (0.17)	-0.002	810 (0.26)	183 (0.31)	-0.010

© 2024 Govier DJ et al. *JAMA Network Open*.

	Tertile 1 (-4, 6)			Tertile 2 (6, 24)			Tertile 3 (24, 187)		
	Comparator, N = 311,963 (83%)	SARS-CoV-2, N = 65,444 (17%)	SMD	Comparator, N = 313,550 (83%)	SARS-CoV-2, N = 63,857 (17%)	SMD	Comparator, N = 317,571 (84%)	SARS-CoV-2, N = 59,835 (16%)	SMD
Transplant	10 (0.00)	0 (0.00)	0.008	384 (0.12)	79 (0.12)	<0.001	2,711 (0.85)	520 (0.87)	-0.002
Stroke/Cerebrovascular Disease	5,047 (1.62)	950 (1.45)	0.014	13,725 (4.38)	2,551 (3.99)	0.019	38,103 (12.00)	7,735 (12.93)	-0.028
Liver Disease	1,128 (0.36)	245 (0.37)	-0.002	27,372 (8.73)	5,954 (9.32)	-0.021	70,096 (22.07)	13,527 (22.61)	-0.013
Kidney Disease	13,397 (4.29)	3,105 (4.74)	-0.022	44,702 (14.26)	9,148 (14.33)	-0.002	152,133 (47.91)	29,698 (49.63)	-0.035
Congestive Heart Failure	496 (0.16)	110 (0.17)	-0.002	11,652 (3.72)	2,322 (3.64)	0.004	83,849 (26.40)	16,807 (28.09)	-0.038
Major Depression Diagnosis	71,005 (22.76)	14,447 (22.08)	0.016	108,310 (34.54)	21,772 (34.09)	0.009	125,347 (39.47)	25,191 (42.10)	-0.054
Anxiety Diagnosis	61,731 (19.79)	12,758 (19.49)	0.007	74,457 (23.75)	14,971 (23.44)	0.007	77,298 (24.34)	15,507 (25.92)	-0.036
PTSD Diagnosis	71,587 (22.95)	14,498 (22.15)	0.019	86,546 (27.60)	17,358 (27.18)	0.009	82,654 (26.03)	16,658 (27.84)	-0.041
Substance Use Disorder	8,549 (2.74)	1,953 (2.98)	-0.015	44,173 (14.09)	8,817 (13.81)	0.008	64,948 (20.45)	13,075 (21.85)	-0.034
Bipolar Diagnosis	6,671 (2.14)	1,331 (2.03)	0.007	12,195 (3.89)	2,251 (3.53)	0.019	17,435 (5.49)	3,740 (6.25)	-0.032
Schizophrenia Diagnosis	217 (0.07)	41 (0.06)	0.003	6,501 (2.07)	961 (1.50)	0.043	14,086 (4.44)	3,179 (5.31)	-0.041
Immunocompromised	13,354 (4.28)	2,879 (4.40)	-0.006	26,665 (8.50)	5,415 (8.48)	0.001	51,879 (16.34)	10,143 (16.95)	-0.017
Community Living Center at Index, n (%)	451 (0.14)	89 (0.14)	0.002	1,195 (0.38)	245 (0.38)	<0.001	6,887 (2.17)	1,559 (2.61)	-0.029
Distance to Nearest VAMC, mean (SD), miles	35.43 (34.83)	34.67 (36.00)	0.021	36.15 (35.17)	35.83 (36.53)	0.009	35.66 (35.22)	34.84 (36.41)	0.023
Non-Preventable Hospitalization Count, 1-year Post-index, mean (SD)	0.07 (0.31)	0.17 (0.50)	-0.257	0.16 (0.52)	0.35 (0.75)	-0.303	0.54 (1.10)	1.02 (1.53)	-0.360
COVID-19 Pandemic Wave of Index, n (%)			0.006			0.001			0.008
First (March-June 2020)	26,559 (8.51)	5,597 (8.55)		28,543 (9.10)	5,812 (9.10)		37,664 (11.86)	7,167 (11.98)	
Second (July-Nov 2020)	111,970 (35.89)	23,671 (36.17)		111,777 (35.65)	22,730 (35.60)		107,760 (33.93)	20,077 (33.55)	
Third (Dec 2020-April 2021)	173,434 (55.59)	36,176 (55.28)		173,230 (55.25)	35,315 (55.30)		172,147 (54.21)	32,591 (54.47)	
Residence in Primary Care Shortage Area, n (%)	76,074 (24.39)	16,549 (25.29)	0.021	74,862 (23.88)	15,802 (24.75)	0.020	74,915 (23.59)	14,796 (24.73)	0.027

© 2024 Govier DJ et al. *JAMA Network Open*.

	Tertile 1 (-4, 6)			Tertile 2 (6, 24)			Tertile 3 (24, 187)		
	**Comparator, N = 311,963 (83%)**	**SARS-CoV-2, N = 65,444 (17%)**	**SMD**	**Comparator, N = 313,550 (83%)**	**SARS-CoV-2, N = 63,857 (17%)**	**SMD**	**Comparator, N = 317,571 (84%)**	**SARS-CoV-2, N = 59,835 (16%)**	**SMD**
Medicare Advantage at Index, n (%)	40,234 (12.90)	7,807 (11.93)	0.029	61,138 (19.50)	12,280 (19.23)	0.007	70,410 (22.17)	15,103 (25.24)	0.072
Long-term Institutionalization During Baseline or Follow-up, n (%)	2,240 (0.72)	653 (1.00)	-0.03	6,960 (2.22)	2,106 (3.30)	-0.066	30,927 (9.74)	8,748 (14.62)	-0.15
Elixhauser Score, mean (SD)	1.05 (2.67)	0.97 (2.72)	0.032	14.51 (5.06)	14.31 (5.03)	0.041	46.85 (19.91)	47.60 (20.81)	-0.037

eTable 9 Footnotes: Pregnancy was a matching variable but was zero for all persons. State of residence was a matching variable and included 50 states and Washington D.C.; Index Month was a matching variable and spanned 14 months (not shown, both SMDs <0.1). Race and ethnicity data from the VA electronic health record are collected through self-identification either at enrollment or at a health care encounter. Abbreviations: SMD: standardized mean difference; CAN: Care Assessment of Need; CDC: Centers for Disease Control and Prevention; VHA: Veterans Health Administration; SD: standard deviation

*Overlapping score categories are mutually exclusive.

© 2024 Govier DJ et al. *JAMA Network Open*.

eTable 10. Sample Characteristics for Veterans With SARS-CoV-2 and Matched Comparators, by Residence in a Primary Care Shortage Area

	Not In Primary Care Shortage Area			Yes, In Primary Care Shortage Area		
	Comparator, N = 717,233 (83%)	SARS-CoV-2, N = 141,989 (17%)	SMD	Comparator, N = 225,851 (83%)	SARS-CoV-2, N = 47,147 (17%)	SMD
**Variables Used in Matching**						
Age Group, n (%)			0.069			0.075
<65	386,663 (53.91)	81,417 (57.34)		120,059 (53.16)	26,816 (56.88)	
65-84	295,385 (41.18)	53,999 (38.03)		95,107 (42.11)	18,193 (38.59)	
85+	35,185 (4.91)	6,573 (4.63)		10,685 (4.73)	2,138 (4.53)	
Sex, n (%)			0.001			0.002
Male	637,120 (88.83)	126,072 (88.79)		202,859 (89.82)	42,323 (89.77)	
Female	80,113 (11.17)	15,917 (11.21)		22,992 (10.18)	4,824 (10.23)	
Race, n (%)			0.022			0.064
American Indian/Alaskan Native	6,086 (0.85)	1,197 (0.84)		2,755 (1.22)	588 (1.25)	
Asian	5,464 (0.76)	1,050 (0.74)		4,158 (1.84)	878 (1.86)	
Black/African American	170,474 (23.77)	34,034 (23.97)		50,089 (22.18)	10,760 (22.82)	
More than one race	7,412 (1.03)	1,426 (1.00)		2,664 (1.18)	604 (1.28)	
Native Hawaiian/Pacific Islander	6,000 (0.84)	1,118 (0.79)		2,856 (1.26)	653 (1.39)	
White	501,340 (69.90)	98,631 (69.46)		154,217 (68.28)	31,203 (66.18)	
Missing	20,457 (2.85)	4,533 (3.19)		9,112 (4.03)	2,461 (5.22)	
Ethnicity, n (%)			0.004			0.047
Hispanic/Latino	61,700 (8.60)	12,055 (8.49)		29,262 (12.96)	6,864 (14.56)	
Not Hispanic/Latino	631,979 (88.11)	125,311 (88.25)		188,343 (83.39)	38,511 (81.68)	
Missing	23,554 (3.28)	4,623 (3.26)		8,246 (3.65)	1,772 (3.76)	
Rurality, n (%)			<0.001			0.057
Urban	502,193 (70.02)	99,444 (70.04)		157,238 (69.62)	34,040 (72.20)	
Not Urban/Missing	215,040 (29.98)	42,545 (29.96)		68,613 (30.38)	13,107 (27.80)	
BMI, mean (SD)	31.34 (6.61)	31.44 (6.33)	-0.017	31.13 (6.60)	31.23 (6.36)	-0.015
Smoking Status, n (%)			0.011			0.037

© 2024 Govier DJ et al. *JAMA Network Open*.

	Not In Primary Care Shortage Area			Yes, In Primary Care Shortage Area		
	**Comparator, N = 717,233 (83%)**	**SARS-CoV-2, N = 141,989 (17%)**	**SMD**	**Comparator, N = 225,851 (83%)**	**SARS-CoV-2, N = 47,147 (17%)**	**SMD**
Never Smoker	284,450 (39.66)	56,799 (40.00)		88,108 (39.01)	18,315 (38.85)	
Current Smoker	91,586 (12.77)	18,126 (12.77)		27,390 (12.13)	5,945 (12.61)	
Former Smoker	302,877 (42.23)	59,282 (41.75)		96,619 (42.78)	19,655 (41.69)	
Missing	38,320 (5.34)	7,782 (5.48)		13,734 (6.08)	3,232 (6.86)	
Nosos Risk-Adjustment Score Category, n (%)*			0.025			0.051
Category 1 (0, 0.417)	17,733 (2.47)	3,832 (2.70)		5,813 (2.57)	1,426 (3.02)	
Category 2 (0.417, 0.471)	31,168 (4.35)	6,521 (4.59)		10,103 (4.47)	2,302 (4.88)	
Category 3 (0.471, 0.534)	42,082 (5.87)	8,528 (6.01)		13,358 (5.91)	2,946 (6.25)	
Category 4 (0.534, 0.611)	52,320 (7.29)	10,430 (7.35)		16,677 (7.38)	3,503 (7.43)	
Category 5 (0.611, 0.707)	62,356 (8.69)	12,168 (8.57)		19,608 (8.68)	4,098 (8.69)	
Category 6 (0.707, 0.829)	73,106 (10.19)	14,253 (10.04)		23,100 (10.23)	4,575 (9.70)	
Category 7 (0.829, 0.998)	83,335 (11.62)	16,141 (11.37)		25,997 (11.51)	5,242 (11.12)	
Category 8 (0.998, 1.259)	94,936 (13.24)	18,470 (13.01)		29,584 (13.10)	5,914 (12.54)	
Category 9 (1.259, 1.805)	109,568 (15.28)	21,382 (15.06)		33,409 (14.79)	6,897 (14.63)	
Category 10 (1.805, 39.370)	133,665 (18.64)	26,990 (19.01)		42,775 (18.94)	9,253 (19.63)	
Missing	16,964 (2.37)	3,274 (2.31)		5,427 (2.40)	991 (2.10)	
CAN score Category, n (%)			0.026			0.048
Category 1 (0, 20)	118,810 (16.57)	24,422 (17.20)		36,052 (15.96)	8,037 (17.05)	
Category 2 (25, 40)	111,275 (15.51)	22,324 (15.72)		34,321 (15.20)	7,217 (15.31)	
Category 3 (45, 60)	135,403 (18.88)	26,334 (18.55)		41,706 (18.47)	8,423 (17.87)	
Category 4 (65, 80)	163,420 (22.78)	31,591 (22.25)		51,268 (22.70)	10,430 (22.12)	
Category 5 (85, 90)	104,983 (14.64)	20,313 (14.31)		34,131 (15.11)	6,921 (14.68)	
Category 6 (95, 99)	69,139 (9.64)	14,201 (10.00)		23,834 (10.55)	5,325 (11.29)	
Missing	14,203 (1.98)	2,804 (1.97)		4,539 (2.01)	794 (1.68)	
Gagne Index Score, mean (SD)	1.40 (2.23)	1.39 (2.31)	0.002	1.40 (2.23)	1.41 (2.33)	-0.005
# VHA Primary Care Visits in Previous 24 Months, mean (SD)	8.30 (10.46)	8.51 (9.65)	-0.021	8.21 (10.21)	8.41 (9.39)	-0.020
# VHA Inpatient Admissions in Previous 24 Months, mean (SD)	0.33 (1.23)	0.34 (1.18)	-0.001	0.39 (1.32)	0.38 (1.24)	0.003
# VHA Specialty Care Visits in Previous 24 Months, mean (SD)	13.46 (15.37)	13.86 (14.10)	-0.027	13.37 (15.24)	13.71 (14.21)	-0.023

© 2024 Govier DJ et al. *JAMA Network Open*.

	Not In Primary Care Shortage Area			Yes, In Primary Care Shortage Area		
	**Comparator, N = 717,233 (83%)**	**SARS-CoV-2, N = 141,989 (17%)**	**SMD**	**Comparator, N = 225,851 (83%)**	**SARS-CoV-2, N = 47,147 (17%)**	**SMD**
COVID-19 Vaccination Before Index Date, n (%)			0.005			0.013
Vaccinated	10,272 (1.43)	2,067 (1.46)		3,258 (1.44)	676 (1.43)	
Not Vaccinated	236,211 (32.93)	47,067 (33.15)		72,844 (32.25)	14,918 (31.64)	
Vaccine Not Available	470,750 (65.63)	92,855 (65.40)		149,749 (66.30)	31,553 (66.92)	
CDC COVID-19 High-Risk Conditions, n (%)						
Cancer	80,926 (11.28)	14,002 (9.86)	0.046	24,983 (11.06)	4,589 (9.73)	0.044
Pulmonary	175,310 (24.44)	33,804 (23.81)	0.015	52,860 (23.40)	10,739 (22.78)	0.015
Hypertension	464,420 (64.75)	90,561 (63.78)	0.020	141,577 (62.69)	28,989 (61.49)	0.025
Diabetes	256,279 (35.73)	49,833 (35.10)	0.013	78,562 (34.78)	16,190 (34.34)	0.009
Dementia	34,804 (4.85)	6,703 (4.72)	0.006	10,674 (4.73)	2,167 (4.60)	0.006
Coronary Heart Disease	204,708 (28.54)	40,032 (28.19)	0.008	61,443 (27.21)	12,416 (26.33)	0.020
Sickle Cell	1,269 (0.18)	271 (0.19)	-0.003	404 (0.18)	93 (0.20)	-0.004
Transplant	2,373 (0.33)	442 (0.31)	0.003	732 (0.32)	157 (0.33)	-0.002
Stroke/Cerebrovascular Disease	43,366 (6.05)	8,439 (5.94)	0.004	13,509 (5.98)	2,797 (5.93)	0.002
Liver Disease	75,046 (10.46)	14,713 (10.36)	0.003	23,550 (10.43)	5,013 (10.63)	-0.007
Kidney Disease	160,422 (22.37)	31,578 (22.24)	0.003	49,810 (22.05)	10,373 (22.00)	0.001
Congestive Heart Failure	72,780 (10.15)	14,406 (10.15)	<0.001	23,217 (10.28)	4,833 (10.25)	0.001
Major Depression Diagnosis	233,359 (32.54)	46,348 (32.64)	-0.002	71,303 (31.57)	15,062 (31.95)	-0.008
Anxiety Diagnosis	164,946 (23.00)	32,837 (23.13)	-0.003	48,540 (21.49)	10,399 (22.06)	-0.014
PTSD Diagnosis	181,818 (25.35)	35,982 (25.34)	<0.001	58,969 (26.11)	12,532 (26.58)	-0.011
Substance Use Disorder	88,153 (12.29)	17,472 (12.31)	<0.001	29,517 (13.07)	6,373 (13.52)	-0.013
Bipolar Diagnosis	27,827 (3.88)	5,525 (3.89)	-0.001	8,474 (3.75)	1,797 (3.81)	-0.003
Schizophrenia Diagnosis	15,689 (2.19)	3,043 (2.14)	0.003	5,115 (2.26)	1,138 (2.41)	-0.010
Immunocompromised	68,299 (9.52)	13,526 (9.53)	<0.001	23,599 (10.45)	4,911 (10.42)	0.001
Community Living Center at Index, n (%)	6,718 (0.94)	1,463 (1.03)	-0.009	1,815 (0.80)	430 (0.91)	-0.012
Distance to Nearest VAMC, mean (SD), miles	36.65 (34.85)	36.51 (36.35)	0.004	32.88 (35.63)	30.94 (35.90)	0.054
**Other Variables (not used in matching)**						

© 2024 Govier DJ et al. *JAMA Network Open*.

	Not In Primary Care Shortage Area			Yes, In Primary Care Shortage Area		
	Comparator, N = 717,233 (83%)	SARS-CoV-2, N = 141,989 (17%)	SMD	Comparator, N = 225,851 (83%)	SARS-CoV-2, N = 47,147 (17%)	SMD
Non-Preventable Hospitalization Count, 1-year Post-index, mean (SD)	0.26 (0.75)	0.50 (1.06)	-0.259	0.26 (0.76)	0.52 (1.11)	-0.28
COVID-19 Pandemic Wave of Index, n (%)			0.003			0.009
First (March-June 2020)	69,797 (9.73)	13,728 (9.67)		22,969 (10.17)	4,848 (10.28)	
Second (July-Nov 2020)	256,003 (35.69)	50,562 (35.61)		75,504 (33.43)	15,916 (33.76)	
Third (Dec 2020-April 2021)	391,433 (54.58)	77,699 (54.72)		127,378 (56.40)	26,383 (55.96)	
Medicare Advantage at Index, n (%)	131,921 (18.39)	26,964 (18.99)	0.015	39,861 (17.65)	8,226 (17.45)	0.005
Long-term Institutionalization During Baseline or Follow-up, n (%)	30,519 (4.26)	8,564 (6.03)	-0.080	9,608 (4.25)	2,943 (6.24)	-0.089
Elixhauser Score, mean (SD)	21.06 (22.77)	20.25 (22.89)	0.035	20.61 (22.45)	20.15 (23.01)	0.020
Elixhauser score tertiles, n (%)*			0.047			0.040
Tertile 1 (-4, 6)	235,889 (32.89)	48,895 (34.44)		76,074 (33.68)	16,549 (35.10)	
Tertile 2 (6, 24)	238,688 (33.28)	48,055 (33.84)		74,862 (33.15)	15,802 (33.52)	
Tertile 3 (24, 187)	242,656 (33.83)	45,039 (31.72)		74,915 (33.17)	14,796 (31.38)	

eTable 10 Footnotes: Pregnancy was a matching variable but was zero for all persons. State of residence was a matching variable and included 50 states and Washington D.C.; Index Month was a matching variable and spanned 14 months (not shown, both SMDs <0.1). Race and ethnicity data from the VA electronic health record are collected through self-identification either at enrollment or at a health care encounter. Abbreviations: SMD: standardized mean difference; CAN: Care Assessment of Need; CDC: Centers for Disease Control and Prevention; VHA: Veterans Health Administration; SD: standard deviation

*Overlapping score categories are mutually exclusive.

© 2024 Govier DJ et al. *JAMA Network Open*.

eTable 11. Sample Characteristics for Veterans With SARS-CoV-2 and Matched Uninfected Comparators, by COVID-19 Pandemic Wave of Index Date (ie, Infection Date)

	**Wave 1 (March to June 2020)**			**Wave 2 (July to November 2020)**			**Wave 3 (December 2020 to April 2021)**		
	**Comparator, N = 92,766 (83%)**	**SARS-CoV-2, N = 18,576 (17%)**	**SMD**	**Comparator, N = 331,507 (83%)**	**SARS-CoV-2, N = 66,478 (17%)**	**SMD**	**Comparator, N = 518,811 (83%)**	**SARS-CoV-2, N = 104,082 (17%)**	**SMD**
**Variables Used in Matching**									
Age Group			0.099			0.068			0.067
<65	48,965 (52.78)	10,708 (57.64)		179,962 (54.29)	38,336 (57.67)		277,795 (53.54)	59,189 (56.87)	
65-84	37,801 (40.75)	6,722 (36.19)		136,088 (41.05)	25,290 (38.04)		216,603 (41.75)	40,180 (38.60)	
85+	6,000 (6.47)	1,146 (6.17)		15,457 (4.66)	2,852 (4.29)		24,413 (4.71)	4,713 (4.53)	
Sex			<0.001			<0.001			0.002
Male	82,704 (89.15)	16,559 (89.14)		295,422 (89.11)	59,242 (89.12)		461,853 (89.02)	92,594 (88.96)	
Female	10,062 (10.85)	2,017 (10.86)		36,085 (10.89)	7,236 (10.88)		56,958 (10.98)	11,488 (11.04)	
Race			0.041			0.031			0.033
American Indian/Alaskan Native	888 (0.96)	170 (0.92)		3,296 (0.99)	681 (1.02)		4,657 (0.90)	934 (0.90)	
Asian	840 (0.91)	173 (0.93)		2,946 (0.89)	597 (0.90)		5,836 (1.12)	1,158 (1.11)	
Black/African American	35,563 (38.34)	7,244 (39.00)		72,913 (21.99)	14,807 (22.27)		112,087 (21.60)	22,743 (21.85)	
More than one race	1,083 (1.17)	205 (1.10)		3,421 (1.03)	694 (1.04)		5,572 (1.07)	1,131 (1.09)	
Native Hawaiian/Pacific Islander	775 (0.84)	161 (0.87)		3,055 (0.92)	601 (0.90)		5,026 (0.97)	1,009 (0.97)	
White	50,745 (54.70)	9,930 (53.46)		235,515 (71.04)	46,676 (70.21)		369,297 (71.18)	73,228 (70.36)	
Missing	2,872 (3.10)	693 (3.73)		10,361 (3.13)	2,422 (3.64)		16,336 (3.15)	3,879 (3.73)	
Ethnicity			0.006			0.017			0.010
Hispanic/Latino	11,187 (12.06)	2,279 (12.27)		32,247 (9.73)	6,805 (10.24)		47,528 (9.16)	9,835 (9.45)	
Not Hispanic/Latino	78,765 (84.91)	15,738 (84.72)		288,780 (87.11)	57,566 (86.59)		452,777 (87.27)	90,518 (86.97)	

© 2024 Govier DJ et al. *JAMA Network Open*.

	Wave 1 (March to June 2020)			Wave 2 (July to November 2020)			Wave 3 (December 2020 to April 2021)		
	**Comparator, N = 92,766 (83%)**	**SARS-CoV-2, N = 18,576 (17%)**	**SMD**	**Comparator, N = 331,507 (83%)**	**SARS-CoV-2, N = 66,478 (17%)**	**SMD**	**Comparator, N = 518,811 (83%)**	**SARS-CoV-2, N = 104,082 (17%)**	**SMD**
Missing	2,814 (3.03)	559 (3.01)		10,480 (3.16)	2,107 (3.17)		18,506 (3.57)	3,729 (3.58)	
Rurality			0.022			0.016			0.012
Urban	77,323 (83.35)	15,636 (84.17)		225,266 (67.95)	45,669 (68.70)		356,842 (68.78)	72,179 (69.35)	
Not Urban/Missing	15,443 (16.65)	2,940 (15.83)		106,241 (32.05)	20,809 (31.30)		161,969 (31.22)	31,903 (30.65)	
BMI	30.86 (6.68)	30.90 (6.45)	-0.007	31.43 (6.63)	31.53 (6.31)	-0.015	31.27 (6.58)	31.39 (6.33)	-0.018
Smoking Status			0.016			0.025			0.015
Never Smoker	37,641 (40.58)	7,615 (40.99)		131,390 (39.63)	26,433 (39.76)		203,527 (39.23)	41,066 (39.46)	
Current Smoker	11,629 (12.54)	2,388 (12.86)		39,811 (12.01)	8,211 (12.35)		67,536 (13.02)	13,472 (12.94)	
Former Smoker	37,182 (40.08)	7,333 (39.48)		143,320 (43.23)	28,143 (42.33)		218,994 (42.21)	43,461 (41.76)	
Missing	6,314 (6.81)	1,240 (6.68)		16,986 (5.12)	3,691 (5.55)		28,754 (5.54)	6,083 (5.84)	
Nosos Risk-Adjustment Score Category, n (%)*			0.029			0.033			0.037
Category 1 (0, 0.417)	4,198 (4.53)	896 (4.82)		10,456 (3.15)	2,313 (3.48)		8,892 (1.71)	2,049 (1.97)	
Category 2 (0.417, 0.471)	3,452 (3.72)	751 (4.04)		14,060 (4.24)	3,065 (4.61)		23,759 (4.58)	5,007 (4.81)	
Category 3 (0.471, 0.534)	4,157 (4.48)	883 (4.75)		19,157 (5.78)	3,992 (6.00)		32,126 (6.19)	6,599 (6.34)	
Category 4 (0.534, 0.611)	5,327 (5.74)	1,094 (5.89)		23,768 (7.17)	4,776 (7.18)		39,902 (7.69)	8,063 (7.75)	
Category 5 (0.611, 0.707)	6,308 (6.80)	1,275 (6.86)		28,731 (8.67)	5,729 (8.62)		46,925 (9.04)	9,262 (8.90)	
Category 6 (0.707, 0.829)	7,915 (8.53)	1,538 (8.28)		33,743 (10.18)	6,594 (9.92)		54,548 (10.51)	10,696 (10.28)	
Category 7 (0.829, 0.998)	9,009 (9.71)	1,782 (9.59)		38,420 (11.59)	7,481 (11.25)		61,903 (11.93)	12,120 (11.64)	
Category 8 (0.998, 1.259)	10,665 (11.50)	2,118 (11.40)		44,449 (13.41)	8,672 (13.04)		69,406 (13.38)	13,594 (13.06)	
Category 9 (1.259, 1.805)	14,315 (15.43)	2,814 (15.15)		50,841 (15.34)	10,002 (15.05)		77,821 (15.00)	15,463 (14.86)	

© 2024 Govier DJ et al. *JAMA Network Open*.

	Wave 1 (March to June 2020)			Wave 2 (July to November 2020)			Wave 3 (December 2020 to April 2021)		
	**Comparator, N = 92,766 (83%)**	**SARS-CoV-2, N = 18,576 (17%)**	**SMD**	**Comparator, N = 331,507 (83%)**	**SARS-CoV-2, N = 66,478 (17%)**	**SMD**	**Comparator, N = 518,811 (83%)**	**SARS-CoV-2, N = 104,082 (17%)**	**SMD**
Category 10 (1.805, 39.370)	24,912 (26.85)	4,927 (26.52)		60,580 (18.27)	12,333 (18.55)		90,948 (17.53)	18,983 (18.24)	
Missing	2,508 (2.70)	498 (2.68)		7,302 (2.20)	1,521 (2.29)		12,581 (2.42)	2,246 (2.16)	
CAN score Category			0.040			0.033			0.033
Category 1 (0, 20)	12,105 (13.05)	2,566 (13.81)		56,865 (17.15)	12,009 (18.06)		85,892 (16.56)	17,884 (17.18)	
Category 2 (25, 40)	12,083 (13.03)	2,522 (13.58)		51,264 (15.46)	10,461 (15.74)		82,249 (15.85)	16,558 (15.91)	
Category 3 (45, 60)	15,368 (16.57)	3,098 (16.68)		63,092 (19.03)	12,313 (18.52)		98,649 (19.01)	19,346 (18.59)	
Category 4 (65, 80)	20,544 (22.15)	4,056 (21.83)		75,095 (22.65)	14,698 (22.11)		119,049 (22.95)	23,267 (22.35)	
Category 5 (85, 90)	16,006 (17.25)	3,027 (16.30)		48,969 (14.77)	9,525 (14.33)		74,139 (14.29)	14,682 (14.11)	
Category 6 (95, 99)	15,162 (16.34)	2,970 (15.99)		30,844 (9.30)	6,438 (9.68)		46,967 (9.05)	10,118 (9.72)	
Missing	1,498 (1.61)	337 (1.81)		5,378 (1.62)	1,034 (1.56)		11,866 (2.29)	2,227 (2.14)	
Gagne Index Score	1.94 (2.68)	1.88 (2.76)	0.021	1.36 (2.18)	1.34 (2.26)	0.008	1.33 (2.16)	1.35 (2.25)	-0.009
# VHA Primary Care Visits in Previous 24 Months, mean (SD)	7.68 (9.89)	7.65 (9.45)	0.004	8.15 (10.21)	8.29 (9.37)	-0.014	8.47 (10.60)	8.75 (9.74)	-0.028
# VHA Inpatient Admissions in Previous 24 Months, mean (SD)	0.58 (1.71)	0.58 (1.63)	0.003	0.33 (1.21)	0.33 (1.15)	0.002	0.31 (1.17)	0.32 (1.13)	-0.003
# VHA Specialty Care Visits in Previous 24 Months, mean (SD)	13.75 (15.85)	13.86 (15.02)	-0.007	13.06 (14.89)	13.36 (13.65)	-0.021	13.63 (15.52)	14.11 (14.26)	-0.032
COVID-19 Vaccination Before Index Date, n (%)			<0.001			<0.001			0.002
Vaccinated	0 (0.00)	0 (0.00)		0 (0.00)	0 (0.00)		13,530 (2.61)	2,743 (2.64)	
Not Vaccinated	0 (0.00)	0 (0.00)		0 (0.00)	0 (0.00)		309,055 (59.57)	61,985 (59.55)	
Vaccine Not Available	92,766 (100.00)	18,576 (100.00)		331,507 (100.00)	66,478 (100.00)		196,226 (37.82)	39,354 (37.81)	

© 2024 Govier DJ et al. *JAMA Network Open*.

	Wave 1 (March to June 2020)			Wave 2 (July to November 2020)			Wave 3 (December 2020 to April 2021)		
	**Comparator, N = 92,766 (83%)**	**SARS-CoV-2, N = 18,576 (17%)**	**SMD**	**Comparator, N = 331,507 (83%)**	**SARS-CoV-2, N = 66,478 (17%)**	**SMD**	**Comparator, N = 518,811 (83%)**	**SARS-CoV-2, N = 104,082 (17%)**	**SMD**
CDC COVID-19 High-Risk Conditions, n (%)									
Cancer	11,939 (12.87)	2,099 (11.30)	0.048	36,182 (10.91)	6,182 (9.30)	0.054	57,788 (11.14)	10,310 (9.91)	0.040
Pulmonary	24,388 (26.29)	4,742 (25.53)	0.017	80,454 (24.27)	15,611 (23.48)	0.018	123,328 (23.77)	24,190 (23.24)	0.012
Hypertension	61,714 (66.53)	12,152 (65.42)	0.023	212,766 (64.18)	41,789 (62.86)	0.027	331,517 (63.90)	65,609 (63.04)	0.018
Diabetes	36,040 (38.85)	7,032 (37.86)	0.020	118,322 (35.69)	23,124 (34.78)	0.019	180,479 (34.79)	35,867 (34.46)	0.007
Dementia	8,821 (9.51)	1,675 (9.02)	0.017	15,601 (4.71)	2,932 (4.41)	0.014	21,056 (4.06)	4,263 (4.10)	-0.002
Coronary Heart Disease	29,751 (32.07)	5,709 (30.73)	0.029	92,941 (28.04)	18,179 (27.35)	0.015	143,459 (27.65)	28,560 (27.44)	0.005
Sickle Cell	281 (0.30)	61 (0.33)	-0.005	554 (0.17)	116 (0.17)	-0.002	838 (0.16)	187 (0.18)	-0.004
Transplant	356 (0.38)	62 (0.33)	0.008	1,129 (0.34)	214 (0.32)	0.003	1,620 (0.31)	323 (0.31)	<0.001
Stroke/Cerebrovascular Disease	7,520 (8.11)	1,464 (7.88)	0.008	19,737 (5.95)	3,828 (5.76)	0.008	29,618 (5.71)	5,944 (5.71)	<0.001
Liver Disease	11,195 (12.07)	2,232 (12.02)	0.002	33,691 (10.16)	6,652 (10.01)	0.005	53,710 (10.35)	10,842 (10.42)	-0.002
Kidney Disease	24,228 (26.12)	4,709 (25.35)	0.018	72,822 (21.97)	14,389 (21.64)	0.008	113,182 (21.82)	22,853 (21.96)	-0.003
Congestive Heart Failure	12,434 (13.40)	2,376 (12.79)	0.018	32,743 (9.88)	6,515 (9.80)	0.003	50,820 (9.80)	10,348 (9.94)	-0.005
Major Depression Diagnosis	33,046 (35.62)	6,611 (35.59)	0.001	105,929 (31.95)	21,304 (32.05)	-0.002	165,687 (31.94)	33,495 (32.18)	-0.005
Anxiety Diagnosis	21,090 (22.73)	4,310 (23.20)	-0.011	74,561 (22.49)	15,098 (22.71)	-0.005	117,835 (22.71)	23,828 (22.89)	-0.004
PTSD Diagnosis	24,890 (26.83)	5,094 (27.42)	-0.013	83,592 (25.22)	16,793 (25.26)	-0.001	132,305 (25.50)	26,627 (25.58)	-0.002
Substance Use Disorder	13,767 (14.84)	2,829 (15.23)	-0.011	39,981 (12.06)	8,156 (12.27)	-0.006	63,922 (12.32)	12,860 (12.36)	-0.001
Bipolar Diagnosis	4,362 (4.70)	863 (4.65)	0.003	11,951 (3.61)	2,409 (3.62)	-0.001	19,988 (3.85)	4,050 (3.89)	-0.002
Schizophrenia Diagnosis	4,043 (4.36)	833 (4.48)	-0.006	6,547 (1.97)	1,284 (1.93)	0.003	10,214 (1.97)	2,064 (1.98)	-0.001

© 2024 Govier DJ et al. *JAMA Network Open*.

	Wave 1 (March to June 2020)			Wave 2 (July to November 2020)			Wave 3 (December 2020 to April 2021)		
	**Comparator, N = 92,766 (83%)**	**SARS-CoV-2, N = 18,576 (17%)**	**SMD**	**Comparator, N = 331,507 (83%)**	**SARS-CoV-2, N = 66,478 (17%)**	**SMD**	**Comparator, N = 518,811 (83%)**	**SARS-CoV-2, N = 104,082 (17%)**	**SMD**
Immunocompromised	10,101 (10.89)	2,028 (10.92)	-0.001	32,678 (9.86)	6,547 (9.85)	<0.001	49,119 (9.47)	9,862 (9.48)	<0.001
Community Living Center at Index	2,429 (2.62)	515 (2.77)	-0.010	2,499 (0.75)	577 (0.87)	-0.013	3,605 (0.69)	801 (0.77)	-0.009
Distance to Nearest VAMC, mean (SD), miles	24.90 (27.50)	24.44 (29.24)	0.016	38.48 (37.35)	37.56 (39.34)	0.024	35.94 (34.41)	35.46 (35.09)	0.014
**Other Variables (not used in matching)**									
Non-Preventable Hospitalization Count, 1-year Post-index, mean (SD)	0.32 (0.87)	0.65 (1.22)	-0.313	0.25 (0.75)	0.49 (1.04)	-0.257	0.25 (0.74)	0.49 (1.07)	-0.260
Primary Care Shortage Area	22,969 (24.76)	4,848 (26.10)	0.031	75,504 (22.78)	15,916 (23.94)	0.028	127,378 (24.55)	26,383 (25.35)	0.018
Medicare Advantage at Index	17,164 (18.50)	3,289 (17.71)	0.021	60,549 (18.26)	12,302 (18.51)	0.006	94,069 (18.13)	19,599 (18.83)	0.018
Hospitalization/Institutional Care, Baseline and Follow-up	5,070 (5.47)	1,634 (8.80)	-0.130	13,960 (4.21)	3,375 (5.08)	-0.041	21,097 (4.07)	6,498 (6.24)	-0.099
Elixhauser Score, mean (SD)	25.36 (25.40)	25.06 (26.53)	0.012	20.65 (22.48)	19.66 (22.40)	0.045	20.35 (22.23)	19.73 (22.45)	0.028
Elixhauser score tertiles, n (%)*			0.043			0.052			0.041
Tertile 1 (-4, 6)	26,559 (28.63)	5,597 (30.13)		111,970 (33.78)	23,671 (35.61)		173,434 (33.43)	36,176 (34.76)	
Tertile 2 (6, 24)	28,543 (30.77)	5,812 (31.29)		111,777 (33.72)	22,730 (34.19)		173,230 (33.39)	35,315 (33.93)	
Tertile 3 (24, 187)	37,664 (40.60)	7,167 (38.58)		107,760 (32.51)	20,077 (30.20)		172,147 (33.18)	32,591 (31.31)	

eTable 11 Footnotes: Pregnancy was a matching variable but was zero for all persons. State of residence was a matching variable and included 50 states and Washington D.C.; Index Month was a matching variable and spanned 14 months (not shown, both SMDs <0.1). Race and ethnicity data from the VA electronic health record are collected through self-identification either at enrollment or at a health care encounter. Abbreviations: SMD: standardized mean difference; CAN: Care Assessment of Need; CDC: Centers for Disease Control and Prevention; VHA: Veterans Health Administration; SD: standard deviation

*Overlapping score categories are mutually exclusive.

© 2024 Govier DJ et al. *JAMA Network Open*.

eTable 12. Sample Characteristics for Veterans With SARS-CoV-2 and Matched Comparators, by Hospitalization at Index

	Not Hospitalized at Index			Yes, Hospitalized at Index		
	**SARS-CoV-2, N = 151,760 (17%)**	**Comparator, N = 756,524 (83%)**	**SMD**	**SARS-CoV-2, N = 37,376 (17%)**	**Comparator, N = 186,560 (83%)**	**SMD**
**Variables Used in Matching**						
Age Group, n (%)			0.191			0.419
<65	96,295 (63.45)	410,762 (54.30)		11,938 (31.94)	95,960 (51.44)	
65-84	50,645 (33.37)	309,244 (40.88)		21,547 (57.65)	81,248 (43.55)	
85+	4,820 (3.18)	36,518 (4.83)		3,891 (10.41)	9,352 (5.01)	
Sex, n (%)			0.002			0.003
Male	132,915 (87.58)	662,989 (87.64)		35,480 (94.93)	176,990 (94.87)	
Female	18,845 (12.42)	93,535 (12.36)		1,896 (5.07)	9,570 (5.13)	
Race, n (%)			0.054			0.092
American Indian/Alaskan Native	1,406 (0.93)	6,973 (0.92)		379 (1.01)	1,868 (1.00)	
Asian	1,646 (1.08)	7,962 (1.05)		282 (0.75)	1,660 (0.89)	
Black/African American	34,706 (22.87)	173,872 (22.98)		10,088 (26.99)	46, 691 (25.03)	
More than one race	1,655 (1.09)	8,145 (1.08)		375 (1.00)	1,931 (1.04)	
Native Hawaiian/Pacific Islander	1,423 (0.94)	7,098 (0.94)		348 (0.93)	1,758 (0.94)	
White	104,577 (68.91)	528,463 (69.85)		25,257 (67.58)	127,094 (68.13)	
Missing	6,347 (4.18)	24,011 (3.17)		647 (1.73)	5,558 (2.98)	
Ethnicity, n (%)			0.039			0.100
Hispanic/Latino	130,520 (86.00)	659,715 (87.20)		33,302 (89.10)	160,607 (86.09)	
Not Hispanic/Latino	15,986 (10.53)	70,965 (9.38)		2,933 (7.85)	19.997 (10.72)	
Missing	5,254 (3.46)	25,844 (3.42)		1,141 (3.05)	5,956 (3.19)	
Rurality, n (%)			0.022			0.016
Urban	107,022 (70.52)	525,967 (69.52)		26,462 (70.80)	133,464 (71.54)	
Not Urban/Missing	44,738 (29.48)	230,557 (30.48)		10,914 (29.20)	53,096 (28.46)	
BMI, mean (SD)	31.42 (6.17)	31.17 (6.53)	0.038	31.28 (6.97)	31.75 (6.92)	-0.067
Smoking Status, n (%)			0.040			0.130
Never Smoker	62,516 (41.19)	298,797 (39.50)		12,598 (33.71)	73,761 (39.54)	
Current Smoker	19,801 (13.05)	97,700 (12.91)		4,270 (11.42)	21,276 (11.40)	

© 2024 Govier DJ et al. *JAMA Network Open*.

	Not Hospitalized at Index			Yes, Hospitalized at Index		
	SARS-CoV-2, N = 151,760 (17%)	Comparator, N = 756,524 (83%)	SMD	SARS-CoV-2, N = 37,376 (17%)	Comparator, N = 186,560 (83%)	SMD
Former Smoker	60,682 (39.99)	316,837 (41.88)		18,255 (48.84)	82,659 (44.31)	
Missing	8,761 (5.77)	43,190 (5.71)		2,253 (6.03)	8,864 (4.75)	
Nosos Risk-Adjustment Score Category, n (%)*			0.071			0.260
Category 1 (0, 0.417)	4,755 (3.13)	19,880 (2.63)		503 (1.35)	3,666 (1.97)	
Category 2 (0.417, 0.471)	7,959 (5.24)	35,357 (4.67)		864 (2.31)	5,914 (3.17)	
Category 3 (0.471, 0.534)	10,309 (6.79)	47,447 (6.27)		1,165 (3.12)	7,993 (4.28)	
Category 4 (0.534, 0.611)	12,339 (8.13)	58,422 (7.72)		1,594 (4.26)	10,575 (5.67)	
Category 5 (0.611, 0.707)	14,200 (9.36)	68,522 (9.06)		2,066 (5.53)	13,442 (7.21)	
Category 6 (0.707, 0.829)	16,184 (10.66)	79,757 (10.54)		2,644 (7.07)	16,449 (8.82)	
Category 7 (0.829, 0.998)	17,993 (11.86)	89,121 (11.78)		3,390 (9.07)	20,211 (10.83)	
Category 8 (0.998, 1.259)	19,879 (13.10)	99,543 (13.16)		4,505 (12.05)	24,977 (13.39)	
Category 9 (1.259, 1.805)	21,666 (14.28)	111,410 (14.73)		6,613 (17.69)	31,567 (16.92)	
Category 10 (1.805, 39.370)	22,695 (14.95)	128,659 (17.01)		13,548 (36.25)	47,781 (25.61)	
Missing	3,781 (2.49)	18,406 (2.43)		473 (1.27)	3,660 (1.96)	
CAN score Category, n (%)			0.117			0.409
Category 1 (0, 20)	30,312 (19.97)	131,384 (17.37)		2,147 (5.74%)	23,478 (12.58)	
Category 2 (25, 40)	26,496 (17.46)	121,857 (16.11)		3,045 (8.15)	23,739 (12.72)	
Category 3 (45, 60)	29,973 (19.75)	145,570 (19.24)		4,784 (12.80)	31,539 (16.91)	
Category 4 (65, 80)	33,491 (22.07)	171,574 (22.68)		8,530 (22.82)	43,114 (23.11)	
Category 5 (85, 90)	18,602 (12.26)	105,916 (14.00)		8,632 (23.10)	33,198 (17.79)	
Category 6 (95, 99)	9,761 (6.43)	65,141 (8.61)		9,765 (26.13)	27,832 (14.92)	
Missing	3,125 (2.06)	15,082 (1.99)		473 (1.27)	3,660 (1.96)	
Gagne Index Score, mean (SD)	1.06 (1.96)	1.29 (2.14)	-0.113	2.76 (3.05)	1.82 (2.54)	0.334
# VHA Primary Care Visits in Previous 24 Months, mean (SD)	7.81 (8.70)	7.92 (10.03)	-0.012	11.22 (12.20)	9.73 (11.67)	0.125
# VHA Inpatient Admissions in Previous 24 Months, mean (SD)	0.22 (0.83)	0.31 (1.17)	-0.092	0.86 (2.02)	0.48 (1.53)	0.213
# VHA Specialty Care Visits in Previous 24 Months, mean (SD)	12.53 (12.70)	12.76 (14.72)	-0.017	19.07 (17.92)	16.20 (17.36)	0.163
COVID-19 Vaccination Before Index Date, n (%)			0.001			0.001

© 2024 Govier DJ et al. *JAMA Network Open*.

	Not Hospitalized at Index			Yes, Hospitalized at Index		
	**SARS-CoV-2, N = 151,760 (17%)**	**Comparator, N = 756,524 (83%)**	**SMD**	**SARS-CoV-2, N = 37,376 (17%)**	**Comparator, N = 186,560 (83%)**	**SMD**
Vaccinated	2,153 (1.42)	10,616 (1.40)		590 (1.58)	2,914 (1.56)	
Not Vaccinated	50,796 (33.47)	253,199 (33.47)		11,189 (29.94)	55,856 (29.94)	
Vaccine Not Available	98,811 (65.11)	492,709 (65.13)		25,597 (68.49)	127,790 (68.50)	
CDC COVID-19 High-Risk Conditions, n (%)						
Cancer	12,634 (8.32)	81,779 (10.81)	-0.085	5,957 (15.94)	24,130 (12.93)	0.086
Pulmonary	30,881 (20.35)	176,766 (23.37)	-0.073	13,662 (36.55)	51,404 (27.55)	0.194
Hypertension	88,575 (58.37)	477,179 (63.08)	-0.097	30,975 (82.87)	128,818 (69.05)	0.328
Diabetes	46,330 (30.53)	258,929 (34.23)	-0.079	19,693 (52.69)	75,912 (40.69)	0.242
Dementia	4,925 (3.25)	32,886 (4.35)	-0.058	3,945 (10.55)	12,592 (6.75)	0.136
Coronary Heart Disease	34,681 (22.85)	204,007 (26.97)	-0.095	17,767 (47.54)	62,144 (33.31)	0.293
Sickle Cell	269 (0.18)	1,312 (0.17)	0.001	95 (0.25)	361 (0.19)	0.013
Transplant	309 (0.20)	2,084 (0.28)	-0.015	290 (0.78)	1,021 (0.55)	0.028
Stroke/Cerebrovascular Disease	6,929 (4.57)	42,755 (5.65)	-0.049	4,307 (11.52)	14,120 (7.57)	0.135
Liver Disease	14,290 (9.42)	75,255 (9.95)	-0.018	5,436 (14.54)	23,341 (12.51)	0.059
Kidney Disease	27,218 (17.93)	159,011 (21.02)	-0.078	14,733 (39.42)	51,221 (27.46)	0.256
Congestive Heart Failure	11,027 (7.27)	70,740 (9.35)	-0.076	8,212 (21.97)	25,257 (13.54)	0.222
Major Depression Diagnosis	49,213 (32.43)	241,154 (31.88)	0.012	12,197 (32.63)	63,508 (34.04)	-0.030
Anxiety Diagnosis	35,767 (23.57)	170,004 (22.47)	0.026	7,469 (19.98)	43,482 (23.31)	-0.081
PTSD Diagnosis	40,200 (26.49)	191,677 (25.34)	0.026	8,314 (22.24)	49,110 (26.32)	-0.095
Substance Use Disorder	18,719 (12.33)	92,278 (12.20)	0.004	5,126 (13.71)	25,392 (13.61)	0.003
Bipolar Diagnosis	5,760 (3.80)	28,945 (3.83)	-0.002	1,562 (4.18)	7,356 (3.94)	0.012
Schizophrenia Diagnosis	2,771 (1.83)	15,874 (2.10)	-0.020	1,410 (3.77)	4,930 (2.64)	0.064
Immunocompromised	12,486 (8.23)	62,250 (8.23)	<0.001	5,951 (15.92)	29,648 (15.89)	0.001
Community Living Center at Index, n (%)	990 (0.65)	5,827 (0.77)	-0.014	903 (2.42)	2,706 (1.45)	0.070
Distance to Nearest VAMC, mean (SD), miles	35.92 (36.58)	36.36 (35.39)	-0.012	31.88 (35.02)	33.27 (33.69)	-0.040
**Other Variables (not used in matching)**						
Non-Preventable Hospitalization Count, 1-year Post-index, mean (SD)	0.32 (0.86)		1.065	0.24 (0.73)		-0.023

© 2024 Govier DJ et al. *JAMA Network Open*.

	Not Hospitalized at Index			Yes, Hospitalized at Index		
	SARS-CoV-2, N = 151,760 (17%)	Comparator, N = 756,524 (83%)	SMD	SARS-CoV-2, N = 37,376 (17%)	Comparator, N = 186,560 (83%)	SMD
COVID-19 Pandemic Wave of Index, n (%)			0.001			<0.001
First (March-June 2020)	13,257 (8.74)	66,210 (8.75)		5,319 (14.23)	26,556 (14.23)	
Second (July-Nov 2020)	53,349 (35.15)	265,962 (35.16)		13,129 (35.13)	65,545 (35.13)	
Third (Dec 2020-April 2021)	85,154 (56.11)	424,352 (56.09)		18,928 (50.64)	94,459 (50.63)	
Medicare Advantage at Index, n (%)	26,471 (17.44)	136,195 (18.00)	0.015	8,719 (23.33)	35,587 (19.08)	0.104
Long-term Institutionalization During Baseline or Follow-up, n (%)	5,062 (3.34)	30,599 (4.04)	-0.038	6,445 (17.24)	9,528 (5.11)	0.393
Elixhauser Score, mean (SD)	16.58 (19.96)	20.00 (22.10)	-0.163	35.04 (27.70)	24.79 (24.59)	0.391
Elixhauser score tertiles, n (%)*			0.155			0.391
Tertile 1 (-4, 6)	60,021 (39.55)	260,529 (34.44)		5,423 (14.51)	51,434 (27.57)	
Tertile 2 (6, 24)	53,490 (35.25)	254,035 (33.58)		10,367 (27.74)	59,515 (31.90)	
Tertile 3 (24, 187)	38,249 (25.20)	241,960 (31.98)		21,586 (57.75)	75,611 (40.53)	

eTable 12 Footnotes: Pregnancy was a matching variable but was zero for all persons. State of residence was a matching variable and included 50 states and Washington D.C.; Index Month was a matching variable and spanned 14 months (not shown, both SMDs <0.1). Race and ethnicity data from the VA electronic health record are collected through self-identification either at enrollment or at a health care encounter. Abbreviations: SMD: standardized mean difference; CAN: Care Assessment of Need; CDC: Centers for Disease Control and Prevention; VHA: Veterans Health Administration; SD: standard deviation

*Overlapping score categories are mutually exclusive.

© 2024 Govier DJ et al. *JAMA Network Open*.
