# Supplementary material for: Risk of Potentially Preventable Hospitalizations After SARS-CoV-2 Infection
Source: JAMA Netw Open. 2024 Apr 10;7(4):e245786. doi: 10.1001/jamanetworkopen.2024.5786 (PMC11007577; doi:10.1001/jamanetworkopen.2024.5786)
Supplement: Supplement 2. — Nonauthor Collaborators [file jamanetwopen-e245786-s002.pdf]

Supplemental Online Content: Nonauthor Collaborators
*First name, last name, and suffix (if applicable) are required and will appear in PubMed.

*Group Name(s): US Department of Veterans Affairs Health Services Research and Development COVID-19 Observational Research Collaboratory							
***First Name and Middle Initial(s)**	***Last Name**	***Suffix (eg, Jr, III)**	**Academic Degrees**	**Institution**	**Location (city, state/province, country)**	**Role or Contribution, eg, chair, principal investigator**	**Group (if more than 1 Group listed in the byline) and/or Subgroup (eg, Steering Committee)**
Theodore	Berkowitz		MS	VA Durham Healthcare System	Durham, NC	Data Manager	
Jacob	Doll		MD	VA Puget Sound Healthcare System	Seattle, WA	Physician Consultant	
Breana	Floyd		MPH	VA Durham Healthcare System	Durham, NC	Research Assistant	
Susan Nicole	Hastings		MD	VA Durham Healthcare System	Durham, NC	Physician Consultant	
Eric	Hawkins		PhD	VA Puget Sound Healthcare System	Seattle, WA	Site Collaborator	
Makoto	Jones		MD	VA Salt Lake City Healthcare System	Salt Lake City, UT	Physician Consultant	
Lee	Kamphius		MPH	VA Ann Arbor Healthcare System	Ann Arbor, MI	Project Manager	
Abby	Moss		BS	VA Portland Healthcare System	Portland, OR	Research Assistant	
Michael	Ong		MD, PhD	VA Greater Los Angeles Healthcare System	Los Angeles, CA	Physician Consultant	
Sarah	Seelye		PhD	VA Ann Arbor Healthcare System	Ann Arbor, MI	Data Analyst	
Javeed	Shah		MD	VA Puget Sound Healthcare System	Seattle, WA	Physician Consultant	
Troy	Shahoumian		PhD	VA Palo Alto Healthcare System	Palo Alto, CA	Data Analyst	
Aasma	Shaukat		MD	VA Minneapolis Healthcare System	Minneapolis, MN	Physician Consultant	
Megan	Banigan		PhD	VA Durham Healthcare System	Durham, NC	Site Collaborator	
Christopher	Slatore		MD	VA Portland Healthcare System	Portland, OR	Physician Consultant	
Battista	Smith		MPH	VA Durham Healthcare System	Durham, NC	Project Manager	
Pradeep	Suri		MD	VA Puget Sound Healthcare System	Seattle, WA	Physician Consultant	
Alan	Teo		MD	VA Portland Healthcare System	Portland, OR	Site Collaborator	
Kelly	Vranas		MD	VA Portland Healthcare System	Portland, OR	Site Collaborator	
Kara	Winchell		MA	VA Portland Healthcare System	Portland, OR	Project Manager	
Edwin	Wong		PhD	VA Puget Sound Healthcare System	Seattle, WA	Site Collaborator	
Kristin	Wyatt		PhD	VA Puget Sound Healthcare System	Seattle, WA	Data Manager	
