# Supplementary material for: Risk of Potentially Preventable Hospitalizations After SARS-CoV-2 Infection
Source: JAMA Netw Open. 2024 Apr 10;7(4):e245786. doi: 10.1001/jamanetworkopen.2024.5786 (PMC11007577; doi:10.1001/jamanetworkopen.2024.5786)
Supplement: Supplement 3. — Data Sharing Statement [file jamanetwopen-e245786-s003.pdf]

Data Sharing Statement

Govier. Risk of Potentially Preventable Hospitalizations After SARS-CoV-2 Infection. *JAMA Netw Open*. Published April 10, 2024. doi:10.1001/jamanetworkopen.2024.5786

Data
Data available: No

Additional Information
Explanation for why data not available: The datasets generated and/or analyzed during the current study are not publicly available due to Department of Veterans Affairs data restrictions prohibiting sharing. Contact the corresponding, Dr. Diana Govier, for data requests.
